# Creation of an Artificial Layer for Boosting Zn^2+^ Mass Transfer and Anode Stability in Aqueous Zinc Metal Batteries

**DOI:** 10.1007/s40820-025-01973-0

**Published:** 2026-01-15

**Authors:** Mingcong Tang, Qun Liu, Gang Liu, Xiaohong Zou, Kouer Zhang, Zhenlu Yu, Biao Zhang, Liang An

**Affiliations:** 1 Department of Mechanical Engineering, The Hong Kong Polytechnic University, Hung Hom, 999077 Hong Kong SAR People’s Republic of China; 2 Department of Applied Physics, The Hong Kong Polytechnic University, Hung Hom, 999077 Hong Kong SAR People’s Republic of China; 3 Research Institute for Smart Energy, The Hong Kong Polytechnic University, Hung Hom, Kowloon, 999077 Hong Kong SAR People’s Republic of China; 4 College of Energy Materials and Chemistry, College of Chemistry and Chemical Engineering, Inner Mongolia University, Hohhot, 010021 People’s Republic of China

**Keywords:** Aqueous zinc metal battery, Artificial layer, Curcumin, Zinc anode

## Abstract

Aqueous zinc metal batteries (AZMBs) are promising candidates for next-generation energy storage, but their commercialization is hindered by zinc anode challenges, notably parasitic reactions and dendrite growth. Herein, we present a biodegradable biomass-derived protective layer, primarily composed of curcumin, as a zincophilic interface for AZMBs. The curcumin-based layer, fabricated via a homogeneous solution process, exhibits strong adhesion, uniform coverage, and robust mechanical integrity. Rich polar functional groups in curcumin facilitate homogeneous Zn^2+^ flux and suppress side reactions. The curcumin-based layer shows a favorable affinity for zinc trifluoromethanesulfonate (Zn(OTf)_2_) electrolyte, which is the representative of organic zinc salts, enabling optimal thickness for both protection and ion transport. The protected Zn anodes demonstrate an extended lifespan of 2500 h in symmetrical cells and a high Coulombic efficiency of 99.15%. Furthermore, Zn(OTf)_2_-based system typically exhibits poor stability at high current densities. Fortunately, the lifespan of symmetrical cells was extended by 40-fold at the high current density. When paired with an NaV_3_O_8_·1.5H_2_O (NVO) cathode, the system achieves 86.5% capacity retention after 3000 cycles at a large specific current density of 10 A g^−1^. These results underscore the efficacy of the curcumin-based protective layer in enhancing the reversibility and stability of metal electrodes, specifically relieving the instability of Zn(OTf)_2_-based systems at high current densities, advancing its commercial viability.

**Supplementary Information:**

The online version contains supplementary material available at 10.1007/s40820-025-01973-0.

## Highlights

- Natural extract, curcumin, was employed as the protective layer to improve the zinc anode stability.
- The curcumin-based layer balanced the efficient thickness, robust adhesion, and facilitated Zn^2+^ transportation.
- The desolvation mechanism with a metal ion chelating agent aside was clearly elucidated.

**Supplementary Information:**

The online version contains supplementary material available at 10.1007/s40820-025-01973-0.

## Graphical abstract

**Figure:**
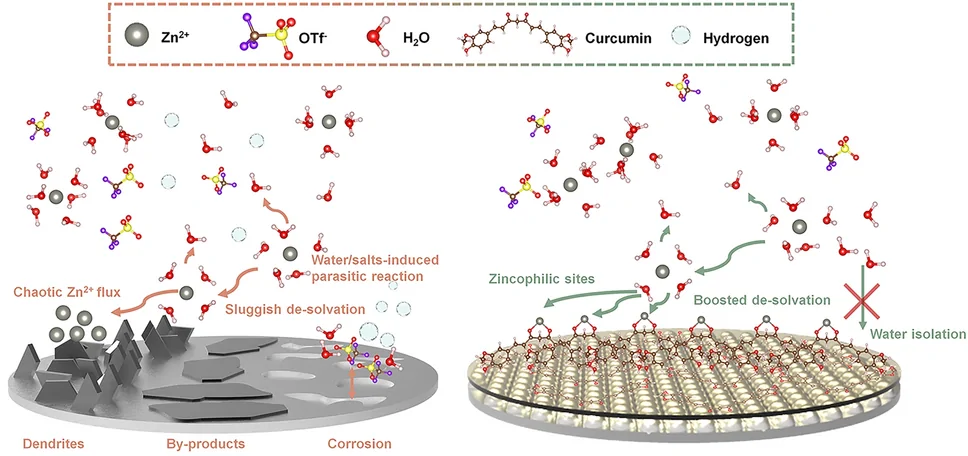

## Introduction

Aqueous zinc metal batteries (AZMBs) are considered crucial participants in next-generation energy storage systems. The growing interest allocated to AZMBs lies in the inherent safety, environmental friendliness, and economic effectiveness of aqueous electrolytes, as well as favorable redox potential (− 0.762 V vs. standard hydrogen electrode), outstanding volumetric capacity (5855 mAh cm^−3^), and massive reserve of zinc anodes [1–3]. However, multiple defects of zinc anodes need to be remedied before the commercialization of AZMBs. Two major challenges are parasitic reactions and dendrite growth, originating from the uncontrollable behavior of water molecules and ions [4–6]. Those side reactions continuously consume the water and salt contents in electrolytes and shadow active areas on the zinc, and the dendrites’ growth will cause the separator penetration [7–9]. Therefore, suppressing parasitic reactions and deriving the homogeneous and flat deposition of zinc are crucial for the stability of AZMBs.

Currently, diverse strategies, including electrolyte engineering [10–12], separator optimization [13–15], and fabrication of surface protective layers [16–18], have been proposed. All of these strategies are converging on two objectives: constructing a robust electrode/electrolyte interphase and guiding the uniform flux of Zn^2+^ [19–21]. Among them, the exertion of an extra protective layer typically provides the most controllable efficacy due to its ex-situ and non-sacrificial features [22, 23]. Depending on the major material in the protective layer, inorganic, organic, and hybrid layers can be chosen [24–26]. The inorganic layers are usually water-insoluble particles and will be cast through a method like the blade coating, together with a minimal binder [27, 28]. The discontinuous characteristic usually results in poor adhesion, and detachment or cracks may happen during bending or cycling [29, 30]. On the other hand, the thickness of the organic layer is sometimes ultra-thin to ensure the Zn^2+^ transportation, resulting in fragile protection [31–33]. The hybrid layer splits the difference, but the phase separation becomes a potential risk [34, 35]. Meanwhile, the selection of protective layers should match the electrolyte system. Zinc sulfide (ZnSO_4_) and zinc trifluoromethanesulfonate (Zn(OTf)_2_) are the two most commonly investigated zinc salts in the current research on aqueous zinc ion battery. The primary advantages of the ZnSO_4_-based electrolytes include the low cost, the high ionic conductivity, and the relatively preferable reversibility. Unique advantages for Zn(OTf)_2_-based system include its compatibility with optimization strategies involving organic contents and its potential as the F-source for the solid electrolyte interphase establishment [36]. However, one extra challenge for the Zn(OTf)_2_ system is its poor stability at high current density and areal capacity. Different constituents in protective coatings may exhibit varying affinity to distinctive electrolytes. Thus, the construction of an artificial homogeneous layer with strong adhesion, suitable inertness, comprehensive zincophilic sites, a non-excessive thickness, and favorable affinity to the electrolyte remains challenging.

**Scheme 1:**
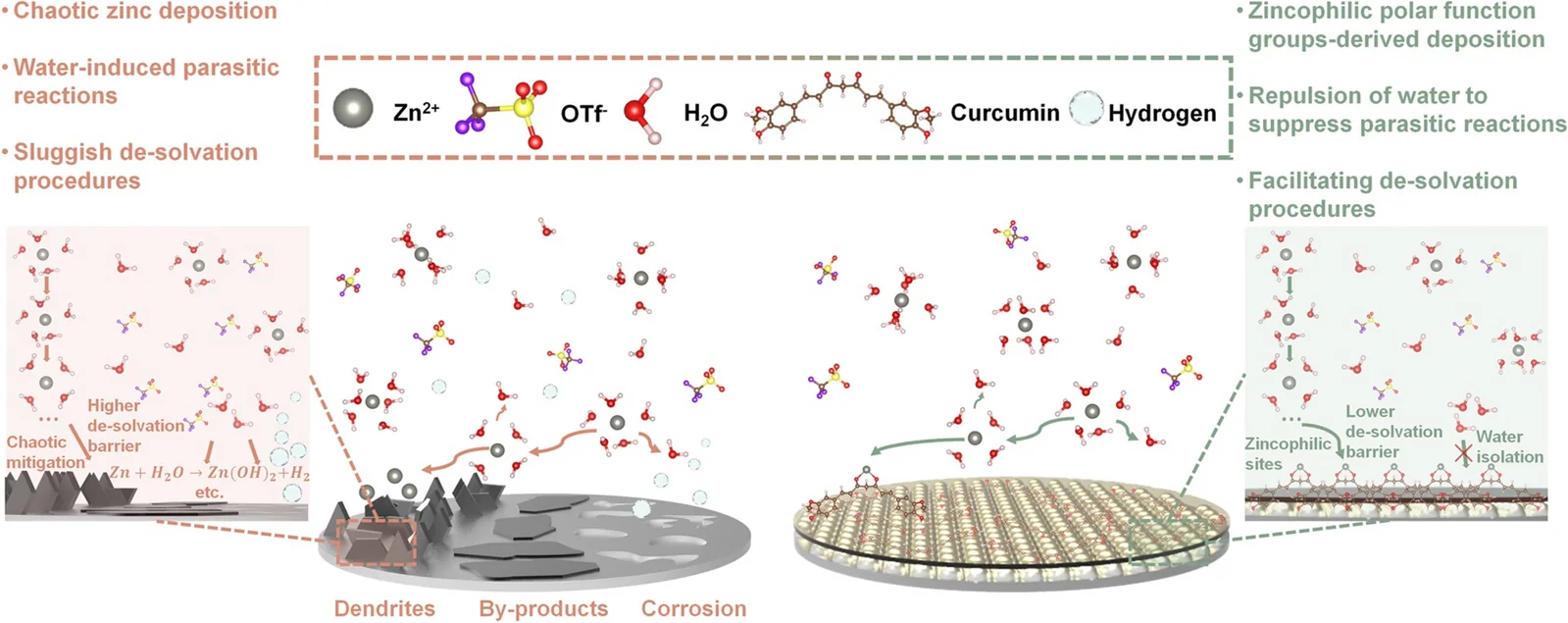
Schematic illustration of efficacy and underlying mechanism of the curcumin artificial protective layer

Herein, we report a biodegradable biomass layer primarily composed of curcumin, a natural extract, as a zincophilic protective layer to achieve a reliable zinc anode. The solubility of curcumin in water, which is only about 1.34 g L^−1^, is very poor, but this value will be thousands-fold in several organic solvents [37]. This sharp contrast makes it a potential active content for the protective layer. As a result, a large proportion of curcumin can be involved during the preparation, and the decomposition of the protective layer can be avoided in the aqueous electrolyte. After dissolving the curcumin into N-methylpyrrolidone (NMP) together with polyvinylidene fluoride (PVDF) as the binder, the precursor is a homogeneous solution instead of a dispersion (Fig. S1), with no precipitation, sediments, or phase separation, so the layer becomes completely uniform and adheres strongly to the substrates. No risk of detachment or break exists under any deformation. Furthermore, rich polar functional groups on the curcumin act as chelating agents, which can anchor Zn^2+^, resulting in the homogeneous and facilitated transportation of charge carriers. Meanwhile, compared to biomasses with an amorphous structure, like lignin or biomasses with a linear structure connecting multiple amorphous units like cellulose, curcumin has a more ordered structure. The symmetrical form with two types of polar functional groups provides a relatively clear binding form. Furthermore, this *β*-diketone structure, which is the structure with its central atoms bonded to two carbonyl groups, usually obtains two forms: the keto form and the enol form (Fig. S2). The keto-enol tautomerism, which means the dynamic equilibrium between these two forms, will happen [38]. Therefore, the coordination of Zn^2+^ on curcumin becomes intuitive. Additionally, within the weakly acidic Zn(OTf)_2_ electrolyte, the keto form tends to dominate, so this self-motivated equilibrium will facilitate the transportation of Zn^2+^. As a result, the overall Zn^2+^ movement kinetics can be boosted. At the same time, this organic layer shows a favorable affinity to organic zinc salts electrolyte (Zn(OTf)_2_ electrolyte), exhibiting a suitable penetration of electrolytes. Therefore, the thickness can be slightly increased to ensure the inertness for anode protection without losing the ion transportation pathways (Scheme 1). Ascribed to these advantages, the curcumin-protected Zn anodes (CUR@Zn) present a superior lifespan up to 2500 h in Zn||Zn symmetrical cells with the Zn(OTf)_2_ electrolyte. The avoidance of parasitic reactions also prevents the waste of zinc and elevates the zinc utilization efficiency. As a result, an extraordinary Coulombic efficiency (CE) of 99.15% was achieved in Zn||Cu cells even after 1200 h of operation. Meanwhile, the lifespan of symmetrical cells was extended by more than 40-fold at the high current density. By coupling with the NaV_3_O_8_·1.5H_2_O (NVO) cathode, the improvement of zinc anode stability was also emphasized in practical full batteries by contributing to a high capacity retention of 86.5% after 3000 cycles with a fast charging/discharging (10 A g^−1^) cycling. All these advancements integrally confirm the virtue of this artificial protective layer for improving the reversibility and stability of zinc anodes, and closing the distance to the commercialization of the Zn(OTf)_2_-based AZMBs by specifically relieving the instability of Zn(OTf)_2_-based systems at high current densities.

## Experimental Section

### Materials

Zinc trifluoromethanesulfonate (98%, Zn(OTf)_2_), curcumin (98%, C_21_H_20_O_6_), and vanadium oxide (99%, V_2_O_5_) were purchased from Macklin. Sodium chloride (99.5%, NaCl) and n-methyl-2-pyrrolidone (98%, NMP) were purchased from Aladdin. Zn foil (0.1 mm thickness), super *P*, and polyvinylidene difluoride (PVDF, HSV-900) were purchased from Kejing Co. Ltd.

### Preparation of the BareZn, CUR@Zn Anode, and the NaV_3_O_8_·1.5H_2_O (NVO) Cathode

#### Preparation of the BareZn and CUR@Zn Anode

The zinc foil was washed in ethanol with ultrasonication for 30 min to remove impurities. Then it was cut into disks with a diameter of 12 mm, denoted as the BareZn. For the CUR@Zn, the curcumin solution was prepared by dissolving the curcumin and PVDF in the NMP with a weight ratio of 9:1. The concentration of the PVDF in NMP is 1.5 wt%. The solution was placed on the heat plate at 80 °C for 4 h under vigorous stirring. Once the solution became orange and uniform, it was cast on the zinc foil with the OSP formed rods. The output thicknesses of the rod are 1 µm, 5 µm, 10 µm, and 15 µm, and the final protected zinc anode was denoted as CUR-1, CUR-5, CUR-10, and CUR-15, respectively. All these curcumin-covered zinc foils were transferred to the vacuum oven and heated up to 80 °C overnight to ensure the complete removal of NMP. Then the foils were cut into disks with a diameter of 12 mm as the anode. After comparing the cycling performance, the final optimal anode was the one fabricated with the 5 µm coater, and was then denoted as the CUR@Zn. Meanwhile, the pure PVDF precursor was also cast to explore the influence of PVDF, and this anode was denoted as PVDF@Zn.

#### Preparation of the NaV_3_O_8_·1.5H_2_O (NVO) Cathode

The synthesis method of the NaV_3_O_8_·1.5H_2_O (NVO) nanobelts powder can be referred to the previous publication [39]. Specifically, 1 g V_2_O_5_ was put into 15 mL 2 M NaCl aqueous solution. The mixture was stirred at 30 °C for 96 h. The red suspension would be collected and washed for 1 time with water and 2 times with ethanol. Then the collected powder was dried in the vacuum oven at 60 °C overnight. The final product was denoted as NVO. The NVO powder was mixed with super P and PVDF with a weight ratio of 7:2:1 in NMP. Then the mixture was mashed in a mortar until a homogeneous slurry was formed. The slurry was cast on the stainless steel foil with a blade coater. After being dried at 80 °C overnight, the cathode was cut into disks and weighed. The estimated loading was 1.5 mg cm^−2^.

More details about materials characterization, electrochemical characterization, simulation, and theoretical calculation are provided in the Supporting Information.

## Results and Discussion

### Electrodes Characterization

**Fig. 1:**
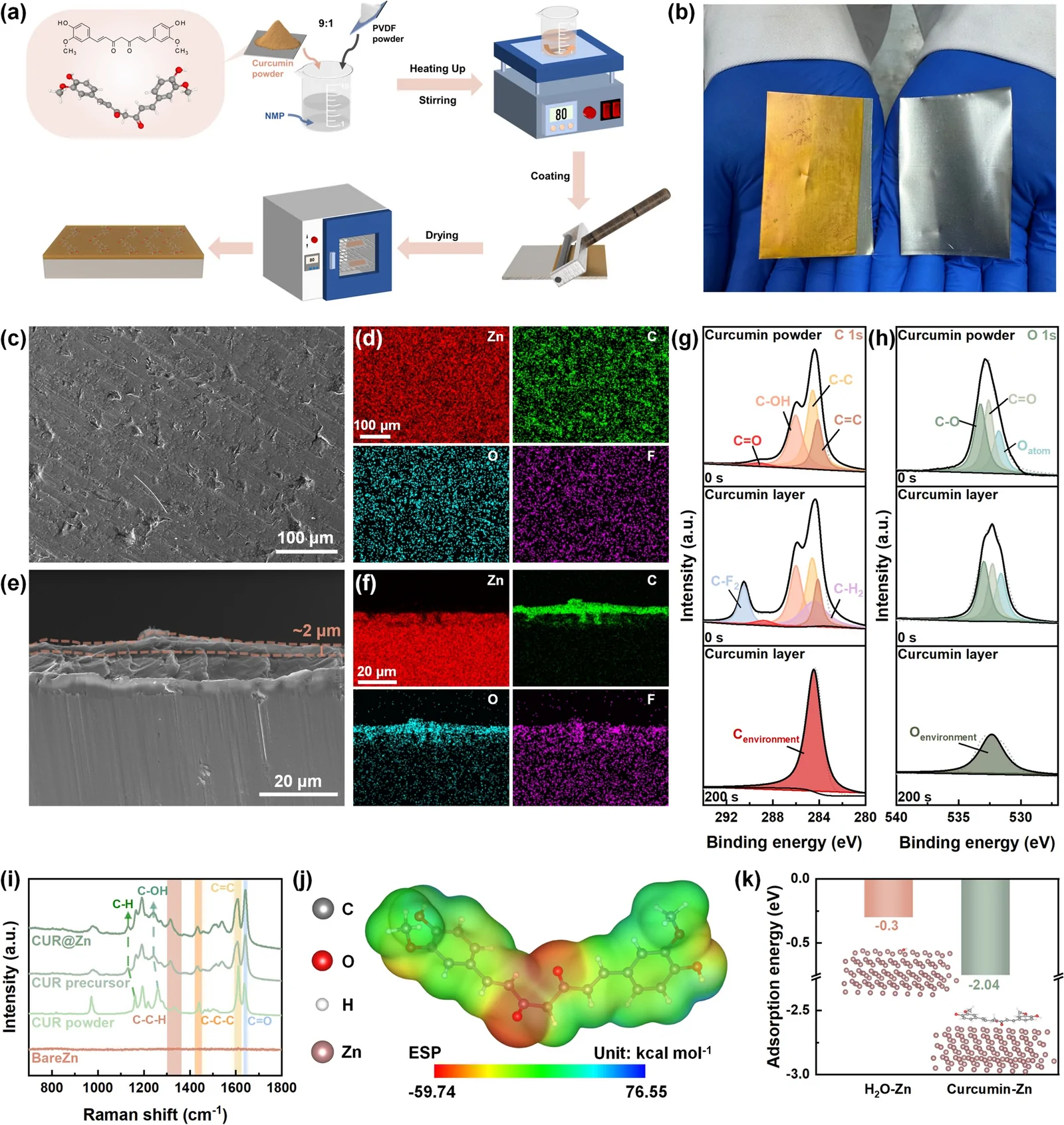
**a** Schematic demonstration of the fabrication procedure of the curcumin protective layer. **b** Digital image of CUR@Zn foils and pristine Zn foils. **c** SEM image of the CUR@Zn foil, and **d** Corresponding elemental mapping image. **e** Cross-sectional SEM image of the CUR@Zn foil, and **f** Corresponding elemental mapping image. The high-resolution XPS spectra of curcumin powder and curcumin layer with different etching times: **g** C 1s, **h** O 1s. **i** Raman spectra of curcumin powder and layer. **j** Electrostatic mapping of the curcumin molecule. **k** Adsorption energies of H_2_O molecule and the curcumin molecule on the Zn(101) plane

The curcumin layer was fabricated through the simple blade coating method by mixing the curcumin powder and PVDF as the binder with a weight ratio of 9:1 to form a homogeneous solution (Fig. 1a). The precursor was then coated on substrates with different thicknesses of the rolling coaters. After being dried overnight, the yellow layer uniformly covered the zinc foil. The optimal thickness of the layer was first determined by obtaining a balance between the ion transport kinetics and the stability improvement. The electrochemical impedance spectroscopy (EIS) of symmetric cells was first utilized to determine the impedance. As illustrated in Fig. S3, with the increasing thickness of curcumin layers, the charge transfer resistance rose remarkably, and when the 10 µm and 15 µm coaters were utilized, the resistance became extremely high. Then, the stability of different anodes was evaluated through symmetrical cells. As shown in Fig. S4, even with the ultra-thin (1 µm coater), the improvement of the zinc anode stability can be observed through the lifespan of about 100 h at 5 mA cm^−2^, and 5 mAh cm^−2^. Meanwhile, the gradual increase in the overpotential shows the same trend with the thickness of the layer. Once the 10 µm coater was employed, even though the cells finally failed at about 300 h, the fluctuation of the voltage signal was obvious due to the tough charge transfer, and the soft-circuit phenomenon occurred. When the coater was increased to 15 µm, the large resistance even caused the cell failure after two cycles. Therefore, the CUR-5 was determined as the optimal anode and denoted as the CUR@Zn.

A series of characterizations and theoretical calculations was carried out to explore the physicochemical properties of the finely tuned curcumin layer. The large weight ratio ensured the comprehensive distribution of curcumin, even with a thin coater. With the 5 μm coater, no omissive area or precursor mitigation was detected. Meanwhile, the thin layer of precursor enabled the fast evaporation of solvent, avoiding the lamination or shift of the layer. As shown in Fig. 1b, there were no cracks or swelling wrinkles on the CUR@Zn foil. Followingly, the mechanical strength of the curcumin layer was demonstrated using the conical indenter. As shown in Fig. S5, the slope of the unload part was used to determine the stiffness and calculate the Young’s modulus. After the calculation, the Young’s modulus of the PVDF layer was about 1.653 GPa, and that of the curcumin layer was about 1.011 GPa. Even though the Young’s modulus decreased slightly, which can be ascribed to the disruption of the intense structure of the PVDF layer because of the large content of curcumin, both values are reasonable for the elastic organic layer. The microstructure and composition of the curcumin layer were first characterized through the scanning electron microscope (SEM) image and corresponding energy-dispersive X-ray (EDX) analysis. The curcumin powder was amorphous rod-like particles with most having an average size of about 5 µm (Fig. S6). After being completely dissolved and cast, as shown in Fig. 1c, the uniformity of the protective layer can be confirmed even under 500 times SEM magnification, and the layer was so thin that the micro-defects on the pristine zinc foils could also be detected. Meanwhile, even though the magnification was increased, defects or pores were not found on the layer (Fig. S7). The surface element mapping also approved the homogeneous distribution of C, O, and F elements on the zinc substrate (Fig. 1d). The comparison of the signal intensities among C, O, and F was consistent with the designed ratio of curcumin and PVDF. The C element, forming the skeleton of both curcumin and PVDF, showed more intense signals than the O elements, which exist on branches. At the same time, the F element was much weaker because only minimal PVDF was added as the binder. The thickness of the layer was determined through the side-view SEM image. As shown in Fig. 1e, the depth of the curcumin layer was about 2 µm, and the energy-dispersive X-ray spectroscopy (EDS) analysis results were perfectly matched with the result of the surface (Fig. 1f). The element concentrations showed similar trends with mapping of the surface, which contains the most C, followed by the O element. Meanwhile, only a minimum F was observed, neglecting the noise signals. The chemical composition of the curcumin layer was further explored through X-ray photoelectron spectroscopy (XPS) measurement. Meanwhile, the same characterization was also carried out for the curcumin powder to confirm elemental constituents. As displayed in Fig. 1g, the high-resolution C 1s spectrum of the curcumin powder showed the presence of C=O, C–OH, C–C, and C=C bonds, which can also be observed on the surface spectrum of the curcumin layer [40]. Furthermore, additional C–F_2_ and C–H_2_ bonds were also detected on the curcumin layer, which can be ascribed to the PVDF binder [41]. Once the etching time was extended to 200 s, all of these signals except environmental C signals disappeared [42], validating the slim thickness. Peaks indicating the C–O and C=O peaks also appeared in the high-resolution O 1s spectrum of both curcumin powder and layer (Fig. 1h). Raman spectroscopy was then carried out. As indicated in Fig. 1i, peaks representing major bonds on curcumin powder, like C=C, C=O, C–OH, etc., were also detected in the curcumin-PVDF precursor solution and the curcumin layer. These peaks show a slight blue shift, reflecting a potential stretch of the bond length, which can be ascribed to the attracting force of the PVDF and NMP. Furthermore, X-ray diffraction (XRD) patterns of the curcumin powder and precursor also showed consistency among components (Fig. S8). Besides experimental evaluation, theoretical calculation based on quantum chemistry and density functional theory (DFT) was executed to investigate the electrochemical property and elucidate the actual active sites of the curcumin. The electrostatic potential mapping (ESP) of the curcumin indicated a higher polarity compared to the water molecule (Fig. S9), and a sharply negative electrostatic potential was exhibited around the carbonyl groups, proving them as more electrophilic reaction sites. Therefore, the coordination of Zn^2+^ mainly happened around the carbonyl groups (Fig. 1j). Additionally, the adsorption energy between the curcumin molecule and the Zn(101) plane, which is the major plane of the zinc foil, as indicated in the XRD result, was also calculated. As presented in Fig. 1k, compared to the H_2_O (− 0.3 eV), an exceptionally more negative adsorption was obtained between the curcumin and Zn (− 2.04 eV). Meanwhile, as shown in Fig. S10, the adsorption energies between other prevalent phases on the zinc foil, which are Zn(002) and Zn(100) planes, are − 1.33 and − 3.43 eV, respectively, both overwhelming the values between H_2_O and the corresponding planes. The elevation of adsorption energies on comprehensive planes suggests the superior adhesion of the curcumin on the Zn surface.

### Kinetics Facilitation

**Fig. 2:**
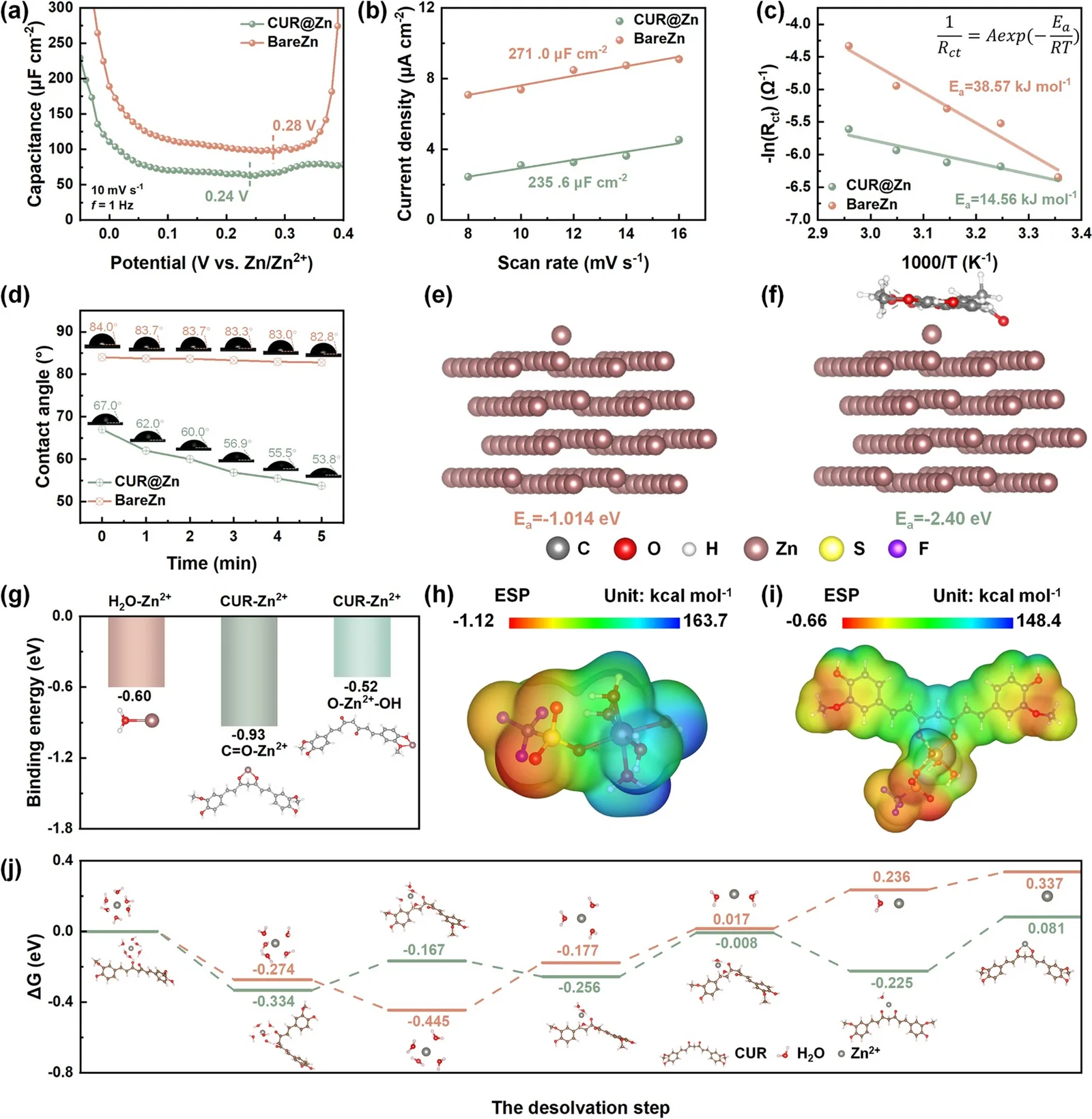
**a** The differential capacitance curve of the Bare Zn anode and the CUR@Zn anode. **b** EDLC for BareZn anode and CUR@Zn anode. **c** Arrhenius curves of different anodes. **d** Contact angles between the electrolyte and different anodes. Adsorption energy of Zn^2+^ on the Zn(101) plane of the **e** BareZn, and **f** CUR@Zn. **g** Binding energies between Zn^2+^ and H_2_O, and different positions on the curcumin molecule. Electrostatic mapping of **h** Zn^2+^-5H_2_O-OTf^−^, and **i** Curcumin-Zn^2+^-4H_2_O-OTf^−^. **j** Profile of Gibbs free energy change for each step of the desolvation process with different anodes

To explore the efficacy of curcumin during the electrochemical reaction, especially for the anodic zinc stripping/plating process, a series of experiments and calculations were carried out to excavate the interfacial mechanism. The influence of the decoration of the curcumin on the interfacial environment was first elucidated through alternating current (AC) voltammetry measurement. As shown in Fig. 2a, the capacitance was decreased on the CUR@Zn surface, and the zero charge potential (PZC) shifted from 0.28 to 0.24 V, suggesting the robust adsorption of the curcumin. Such interfacial adsorption behavior was further supported by the decrease in electric double-layer capacitance (EDLC) from 271.0 to 235.6 µF cm^−2^ (Figs. 2b and S11). These strongly adsorbed curcumin molecules could effectively isolate water molecules from the adsorption, avoiding the water-induced parasitic reactions. Besides the successful adhesion, the efficacy in regulating the Zn^2+^ transportation kinetics was then evaluated. The charge transfer resistance for symmetrical cells with BareZn and CUR@Zn anodes was measured (Fig. S12), and then the relationship between the inverse of temperature and the opposite number of the natural logarithm of the charge transfer resistance (− ln(*R*_ct_)) was obtained [43]. As shown in Fig. 2c, the slope after the linear calibration was used to determine the activation energy for the cells with CUR@Zn as 14.56 kJ mol^−1^, which was much smaller than its counterpart with BareZn (38.57 kJ mol^−1^). This reduced activation energy effectively confirmed the facilitated interfacial Zn^2+^ transportation kinetics. Moreover, the Zn^2+^ transference number (*t*_Zn2+_) was also assessed by measuring the EIS before and after the reaction with a constant potential. With the curcumin layer, the *t*_Zn2+_ was increased from 0.383 to 0.435, also proving a boosted ion movement (Fig. S13). The electrode wettability was then evaluated by measuring contact angles. As displayed in Fig. 2d, contact angles between electrolytes and zinc anodes were remarkably lowered from 84° to 67° with the curcumin layer. Meanwhile, contact angles showed negligible variation on BareZn for 5 min, but a consistent decrease from 67° to 53.8° on CUR@Zn foils. Furthermore, as illustrated in Fig. S14, the contact angle on the PVDF@Zn was 105.51°, and only a minimum decrease to 104.33° was exhibited in 5 min, demonstrating the hydrophobic property of the PVDF. Therefore, it was difficult for the electrolyte to penetrate the PVDF layer, and the ionic conduction would be difficult. The result confirmed the improved affinity of the curcumin layer to electrolytes, which could be ascribed to the presence of hydrophilic polar functional groups (C=O). This favorable wettability was effective for reducing the interfacial free energy at the electrode/electrolyte interface, contributing to the homogeneous electrodeposition.

Additionally, the adsorption energies (*E*_*a*_) of Zn^2+^ on the BareZn(101) and CUR@Zn(101) were calculated based on DFT. As indicated in Fig. 2e, f, with the curcumin layer, *E*_*a*_ on Zn(101) was elevated from − 1.01 to − 2.40 eV, suggesting that the curcumin was effective for expediting the transportation process of Zn^2+^ from the electrolyte to the Zn(101) surface. Adsorption energies of Zn^2+^ on the other two planes of BareZn and CUR@Zn were also calculated. With the curcumin layer, the adsorption energies of Zn^2+^ on Zn(002) and Zn(100) planes were increased from − 0.5 to − 2.49 eV, and from − 2.59 to − 4.85 eV, respectively (Fig. S15). Therefore, the facilitation of Zn^2+^ on prevalent planes of zinc foils with the presence of curcumin can be confirmed. This promoted process implied the capability of a more uniform and compact Zn conduction pattern, inhibiting dendrite formation. To elucidate the actual complex structure, binding energies between curcumin and Zn^2+^ were then calculated. Referring to previous ESP results, two positions, including C=O groups and -OH groups, were chosen as potential binding sites. Meanwhile, the binding energy between the PVDF and Zn^2+^ was also calculated to verify the actual source of boosted ion transportation kinetics. As shown in Fig. 2g, the binding energy between C=O and Zn^2+^ reached − 0.93 eV, which overwhelms that between H_2_O and Zn^2+^, which was only − 0.6 eV. The binding energy between the –OH group, which is another potential anchor, and Zn^2+^ was also calculated, and a reduced value (− 0.52 eV) was obtained. Furthermore, the binding energies between both polar groups and Zn^2+^ were much larger compared to that between the PVDF and Zn^2+^, which was − 0.145 eV (Fig. S16). Thus, the preference for Zn^2+^ to be bound with the curcumin molecule on the C=O group can be confirmed. Raman spectroscopy and Fourier transform infrared spectroscopy (FTIR) were also used to confirm the binding structure. As shown in Fig. S17a, after cycling for 30 cycles, one new peak emerged at about 165 cm^−1^, which can be ascribed to the Zn–O bond [44]. Similar variation can also be observed in the FTIR pattern (Fig. S17b). One FTIR signal at the wavenumber of 450 cm^−1^ was detected, which corresponded to the stretching vibration of Zn–O [45]. The XPS was also utilized to identify the surface elemental composition after cycling. As illustrated in Fig. S18, the high-resolution C 1s spectrum still showed the presence of C=O, C–OH, C–C, and C=C bonds, which was consistent with the curcumin layer. Meanwhile, the combination of O 1s and Zn 2p spectra verified the existence of a Zn–O bond. Furthermore, in the Raman spectrum, major bonds, including C=C, C=O, C–H, etc., were preserved, and in the FTIR spectrum, signals indicating the vibration of bonds on the curcumin were exhibited with a slight shift. Combining variations shown in Raman, FTIR spectrum, and XPS analysis, the electrochemical stability of the curcumin structure was demonstrated, and the dominating adsorption form would be C=O–Zn^2+^, which was chosen as the primary structure for further theoretical calculations.

The variation of ESP once the binding between the curcumin molecule and Zn^2+^ was then calculated, and the electrostatic potential showed an extensive reduction for the whole structure (Fig. 2h, i). This more negative ESP value indicates a cutback of the electrostatic repulsion around the Zn^2+^, which results in a more facilitating ion transportation [46]. To further reveal the dominating binding structure, the ESP when Zn^2+^ was bound to the –OH group was also calculated. As shown in Fig. S19, the electrostatic potential around the C=O groups still presented the most negative value, further confirming that the preferable structure was C=O–Zn^2+^. Combining all these merits, the curcumin layer was expected to improve the desolvation kinetics, so the Gibbs free energy change profiles for stepwise desolvation procedures of the [Zn(H_2_O)_6_]^2+^ structure without/with the close presence of curcumin were obtained. As indicated in Fig. 2j, on the BareZn anodes, only the trimming steps of the first two water molecules were intuitive, and all following steps required much external energy input to motivate the exclusion of H_2_O. On the contrary, three water molecules could be thrown out of the sheath with the self-motivation once they approached the curcumin molecule. Meanwhile, for the eradication of all water molecules, the overall required energy input was also significantly reduced on the CUR@Zn, confirming the facilitation of the desolvation process. Meanwhile, the Gibbs free energy change for the desolvation process, assuming the curcumin molecule participated directly in the solvation sheath, was also calculated. Ascribed to the strong binding force between the curcumin and Zn^2+^, two water molecules could be replaced. As shown in Fig. S20, the extraction of the first two H_2_O molecules was still intuitive. Even though the external energy input was required for the complete desolvation of the additional two, the overall energy input was still decreased, so the desolvation process could also be facilitated.

### Parasitic Reaction Suppression

**Fig. 3:**
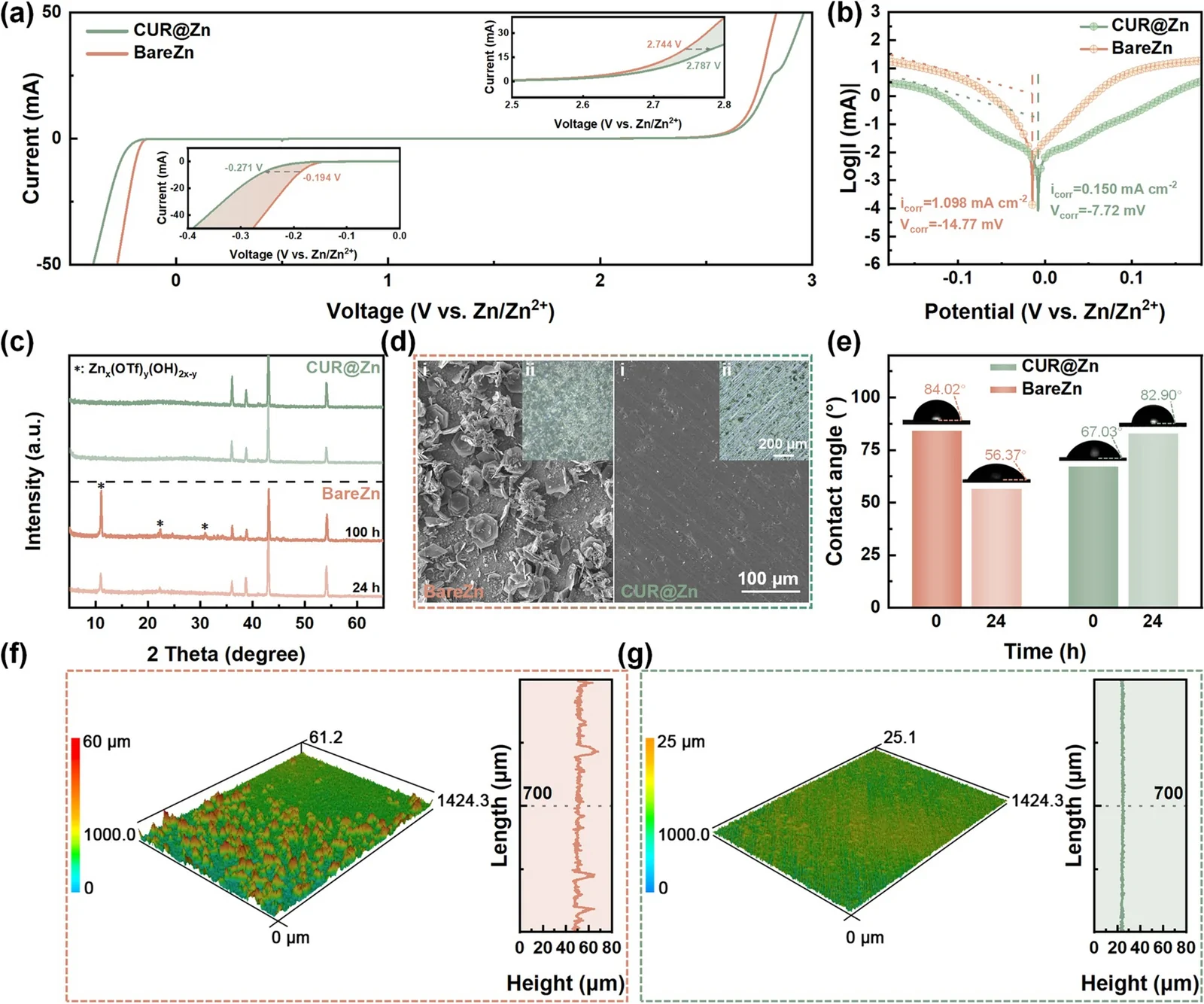
**a** Overall electrochemical stability window of different anodes, insets show the HER and OER potentials. **b** Linear polarization curves of BareZn anode and CUR@Zn anode. **c** XRD patterns of different anodes soaked in the electrolyte for 24 h and 100 h. **d i** SEM images and **ii** optical microscopic images of BareZn and CUR@Zn soaked in electrolytes for 100 h. **e** Contact angles between the electrolyte and pristine BareZn and CUR@Zn anode, and between the electrolyte and different anodes after corrosion. 3D topology and corresponding height profile obtained by CLSM analysis of **f** BareZn and **g** CUR@Zn after corrosion

The inherent chemical inertness, improved affinity of Zn^2+^, and the boosted Zn^2+^ transportation kinetics of the curcumin layer show potential for the suppression of both the electrochemical and chemical parasitic reactions. With the aqueous electrolyte, the major competing reaction during the cycling is water electrolysis. Therefore, the linear sweep voltammetry (LSV) test was first carried out to measure the potentials of the hydrogen evolution reaction (HER) and oxygen evolution reaction (OER) for determining the electrochemical stability window (ESW). As shown in Fig. 3a, with the BareZn anode, the HER and OER happened at – 0.194 V and 2.744 V, respectively. The curcumin layer provided a 0.12 V wider ESW with an outstanding decrease of the potential of HER to – 0.271 V. Meanwhile, with the reduced interfacial activity, the OER potential was also slightly increased to 2.787 V. The range of the LSV test was then shrunk to the non-Faradaic region to exclude electrochemical reactions. The corrosion current and voltage were obtained through the extrapolation of the logarithm of current signals. The corrosion current indicates the rate of corrosion. The corrosion voltage is the potential at which the corrosion happens, and it can be used to describe the tendency for corrosion will occur [47]. As shown in Fig. 3b, the curcumin layer lowered the corrosion current from 1.098 to 0.15 mA cm^−2^, elucidating an extremely slow corrosion rate. Meanwhile, a positive shift in the corrosion voltage from – 14.77 to – 7.72 mV can be observed. This more positive corrosion voltage confirms the increase in the interfacial inertness to chemical corrosion for the CUR@Zn. Combining with the decrease in corrosion current density, both the weaker intention to be corroded and a much slower corrosion rate were exhibited.

The corroboration of the improved corrosion resistance capability was then executed through a series of optical observations and semi-quantitative analysis of eroded anodes. Both electrodes were soaked in the designed Zn(OTf)_2_-based electrolytes, and the duration of treatment ranged from 24 to 100 h. The XRD spectroscopy was utilized to determine surface components. As indicated in Fig. 3c, peaks representing corrosion products can be found at around 12°, 23°, and 30° on BareZn after soaking for 24 h. The peaks were even more intense for longer treatment. On the contrary, no unexpected peaks appeared on the CUR@Zn anode from 24 to 100 h of corrosion. The XRD pattern was kept consistent with pristine zinc foils (Fig. S21). Furthermore, the Raman spectroscopy was used to precisely determine the accumulation of amorphous corrosion products. As shown in Fig. S22, peaks attributed to ZnO, and Zn(OH)_2_ became intense for BareZn. On the contrary, no similar peaks were found on the CUR@Zn anode, which is consistent with the pristine zinc foil. The high-resolution optical and SEM images were also taken on these anodes. For the BareZn, dark gray by-products were shown in the optical images and almost covered the whole electrode. When these areas were further magnified in the SEM image, the intensive sheet-like products with an average size of about 50 µm were detected (Fig. 3d). Meanwhile, no such undesired products were found in either optical or SEM images of CUR@Zn anodes, and the flawless and shiny surface was kept, just the same as untreated zinc foils (Fig. S23). The variation of the hydropathy property was subsequently evaluated. The contact angles of the BareZn and CUR@Zn were measured without any corrosion behavior. As shown in Fig. 3e, the contact angle of the electrolyte on the BareZn is 84.02°, and it was decreased to 67.03° on the CUR@Zn, confirming the zincophilicity of the curcumin layer, which is consistent with previous characterizations and theoretical calculations. Then, both foils were immersed for 24 h, and the contact angles were tested again. For the CUR@Zn, the curcumin layer was removed before the test to quantify the generation of by-products on the zinc foil. After corrosion, the contact angle on the BareZn was sharply decreased to 56.37°, which was ascribed to the widespread by-products. These water-induced by-products contain rich hydroxyl groups, which are intrinsically hydrophilic [48]. Instead, the contact angle on the CUR@Zn foil was recovered to 82.9°, once the curcumin layer was removed. The value is almost the same as the contact angle on the unpolluted zinc foil, demonstrating the unchanged surface environment. The confocal laser scanning microscope (CLSM) was then employed to depict the macroscopic roughness of different anodes. As demonstrated in Fig. 3f, abundant juts with a height of about 60 µm were widely spread on the BareZn after electrolyte treatment. The surface profile also showed repeated elevation of the surface height. Contrarily, negligible undulation was found on the CUR@Zn, and the surface elevation was kept stably at about 21 µm (Fig. 3g), which was consistent with uncorroded zinc foils (Fig. S24). Both the reconstruction of surface morphology and the surface profile showed no difference from those of the original zinc anodes. Therefore, the improved surface inertness and preferable selectivity of Zn^2+^ successfully suppressed the interfacial water-induced parasitic reactions. Meanwhile, the isolation of electrodes from the electrolytes effectively avoided the chemical etching.

### Deposition Behavior

**Fig. 4:**
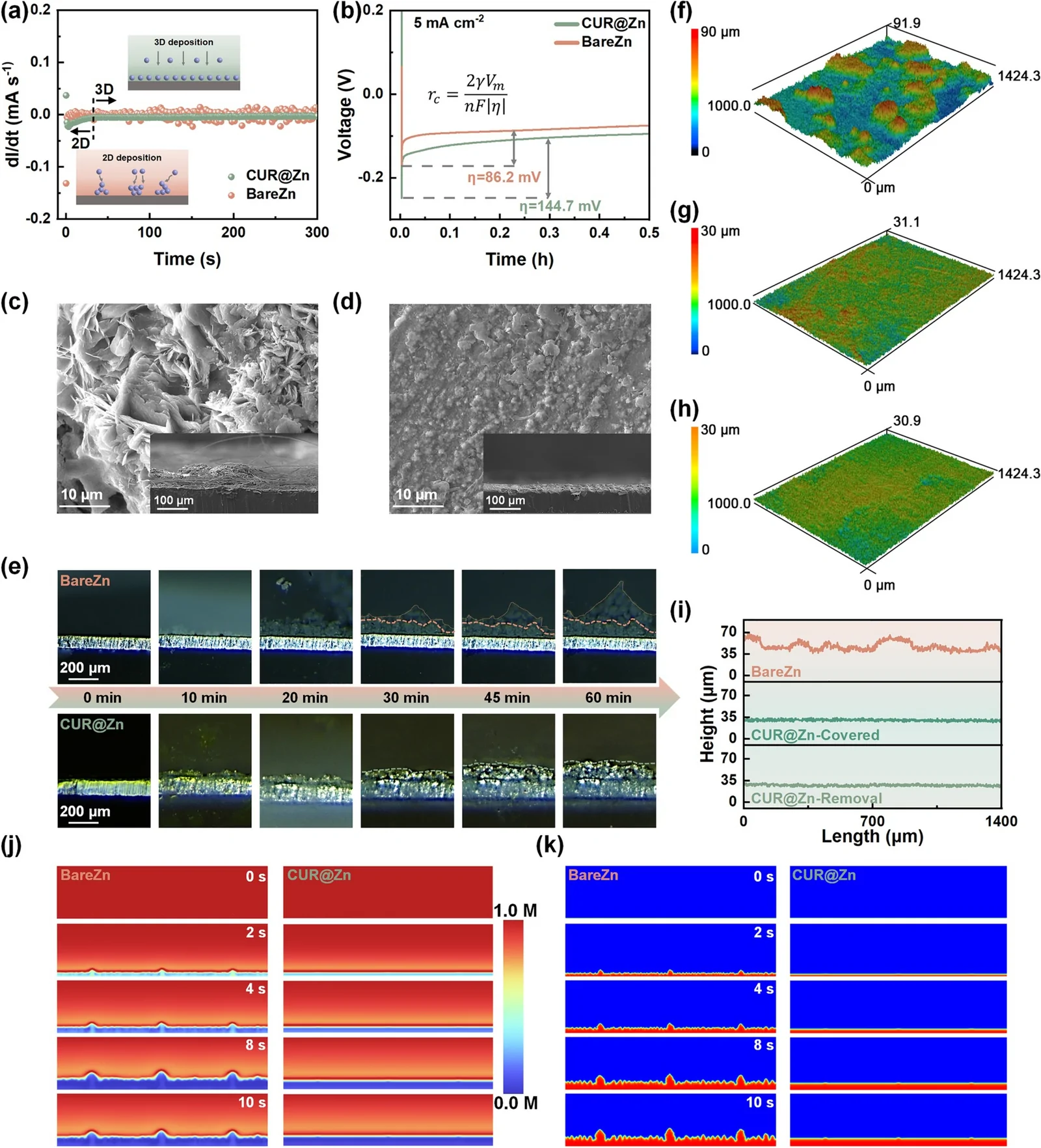
**a** dI/dt curves for symmetrical cells using BareZn and CUR@Zn. **b** The initial nucleation overpotential using different anodes in Zn||Cu cells. SEM images and cross-sectional SEM images of **c** BareZn and **d** CUR@Zn after cycling. **e** In situ optical microscope observation of Zn deposition behavior on BareZn and CUR@Zn. 3D topology obtained by CLSM analysis of **f** BareZn, **g** CUR@Zn with curcumin layer covered, and **h** CUR@Zn with curcumin layer removed after cycling. **i** Corresponding height profiles of different anodes. COMSOL simulation results of **j** ion distribution and **k** dendrite growth on different anodes

With the boosted Zn^2+^ transportation and uniform Zn^2+^ flux derived by zincophilic functional groups on the curcumin, ameliorated deposition behavior can be elicited. The deposition mode was first appraised through the chronoamperometry (CA) test. The time derivative was recorded with a constant voltage bias of – 0.25 V. As indicated in Fig. 4a, for the BareZn anode, the current fluctuated for all 300 s. This indicates a 2D deposition as shown in the inset scheme. The reduction of Zn^2+^ cannot happen immediately once it arrives at the surface of the electrode. Instead, these ions continuously migrate along the zinc foil and then aggregate according to the non-uniform distribution of the local electric field. As a result, the dendrite is formed and grows [49]. Meanwhile, the continuous corrosion and passivation caused the defects and loss of active areas. This precarious operation led to the ceaseless surface area variation, so the current also oscillated when the voltage was constant. On the contrary, the curcumin layer regulates the Zn^2+^ flux and avoids chaotic navigation. As a result, the ordered 3D deposition happened, causing no surface area variation. Thus, the current signal became stable within 30 s. The nucleation overpotential for the first cycle was then measured to determine the nucleation pattern. Because only the deposition for the first cycle was considered to elucidate the nucleation behavior, the Zn||Cu half-cell with the Cu covered with the curcumin layer was utilized. A constant current density of 5 mA cm^−2^ was employed to motivate the zinc deposition on the Cu substrate. As shown in Fig. 4b, the curcumin layer increased the nucleation overpotential from 86.2 to 144.7 mV. This increasing trend of the nucleation overpotential could also be observed for the first deposition among all current ranges (Fig. S25**)**. According to the classical nucleation theory, only a nucleus with a particle size larger than the critical radius can be thermodynamically stable. The critical radius is found when the changes in the volume energy and surface energy reach the equilibrium, which means that the first derivative of the total Gibbs free energy ($\Delta {G}_{T}$) as shown in Eq. 1 is zero [50]:

1 $$\Delta {G}_{T}=-\frac{4}{3}\pi {r}^{3}\Delta {g}_{v}+4\pi {r}^{2}\gamma$$

Then the critical radius can be deduced as Eq. 2, where γ is the surface tension required to be broken to form the nuclei, ${V}_{m}$ is the molar volume, n is the charge number, and F is the Faraday’s constant.

2 $${r}_{c}=\frac{2\gamma }{\left|\Delta {g}_{v}\right|}=\frac{2\gamma {V}_{m}}{nF\left|\eta \right|}$$

In this equation, *n* and *F* are constants. γ and ${V}_{m}$ can be viewed as constants for the same electrolyte and the interface where the deposition happens. Therefore, this equation implies that the critical radius is inversely proportional to the nucleation overpotential. Therefore, the curcumin layer brought the smaller nucleus that distributed more intensively and uniformly. As shown in Fig. S26, for the first deposition, with the curcumin layer, the surface was fully covered with nuclei less than 0.5 μm. Even for the zinc growing for some time, the size is only about 5 μm. On the contrary, large aggregates and inactive areas can be found on the BareZn. The size of the largest particle even exceeds 50 μm. If the accretion of these particles continues, a macroscopic dendrite will be formed. The morphology evolution for continuous cycling was also verified from a series of optical spectroscopies. The SEM images of zinc anodes cycling after 10 cycles at 1 mA cm^−2^ were taken. The low-magnification figures showed that aggregation was intensive on the BareZn anode, but the zinc deposited on the CUR@Zn was flat and lateral flakes that formed no tips (Fig. S27). Once the surface morphology was observed with a further amplified view, as shown in Fig. 4c, protrusions with a height of about 100 µm can be detected from the cross-section of the zinc foil, and the magnified picture of the surface shows the chaotic growth of zinc with aggregated morphology and spiculate dendrites. In sharp contrast, the surface of the CUR@Zn anode was kept extremely smooth with a homogeneous thickness (Fig. 4d). This remarkable difference happened even for the first cycle and became increasingly intense after the longer operation (Fig. S28). To further elucidate the actual deposition process, the in situ optical observation was carried out on the side views of different electrolytes. To exhibit the sediment of zinc more clearly, the amount of deposition was chosen as 10 mAh cm^−2^. As shown in Fig. 4e, for the BareZn anode, zinc aggregation can be observed after depositing for 10 min, and the shadow of uneven deposition and corrosion products in the depth of the zinc foil. With the extended deposition duration, the surface became jaggy with an obvious height difference between tips and defects. Meanwhile, the accumulation of loose parasitic reactions and the shadow of even more protruding zinc can be detected after 1 h. The nucleation sites are more uniform with the curcumin layer, as shown in the first 10 min. Meanwhile, the growth of zinc was kept homogeneous for the whole 60 min, and no loose by-products emerged. The deposition behavior will directly influence the roughness of the surface. Thus, a CLSM was utilized to quantify the degree of crudeness of these anodes after cycling for 30 cycles. The reconstruction of zinc anodes without the curcumin layer showed a large surface variation between 10 and 90 µm, and the most protuberant tip reached the height of 91.9 µm (Fig. 4f). Conversely, the loftiness of the curcumin-covered zinc anode was restricted to about 30 µm, with the highest point of 31.1 µm. Once the layer was removed, the flat surface could be kept with a uniform elevation distribution (Fig. 4g, h). The height variation can be identified more clearly from the profile of a horizontal line on different anodes (Fig. 4i). Therefore, the curcumin layer successfully derived the uniform deposition of zinc, facilitating the construction of a flat surface. As a result, the concern of separator penetration caused by bulges can be relieved. The influence of the curcumin layer on the ion concentration distribution and corresponding deposition behavior was reproduced via the COMSOL Multiphysics module with parameters from previous publications and measured values (Table S1), for a more intuitive demonstration. As shown in Fig. 4j, the curcumin layer converts the distribution of Zn^2+^ from the anisotropic dispersion to an isotropic mode. This means that Zn^2+^ ions stop aggregating around the tips to evoke the ‘tip effects’. Instead, ions distribute more evenly over the whole electrode, promising a relatively fair possibility of deposition on all active sites. Consequently, uneven nucleation can be avoided at the initial stage, and no dendrites are detected for the prolonged deposition with the CUR@Zn anode (Fig. 4k).

### Cells Performance

By regulating the electric double layer, contributing a stronger binding force between Zn^2+^, and facilitating the desolvation process, the curcumin protective layer shows effectiveness for a more uniform and effective deposition behavior. By combining its efficacy in preventing corrosion, the stability of the zinc anode is expected to be improved. Thus, the lifespan of symmetrical cells was first evaluated with the CUR@Zn as electrodes. To exclude the influence of the PVDF binder, the symmetrical cells using PVDF@Zn were also tested. The curcumin was removed from the precursor, and the PVDF was coated on the zinc foils directly using the device with the same thickness. As shown in Fig. S29, the stability of the zinc anode was not improved, and the inertness of the PVDF layer caused an obvious increase in the overpotential ascribed to the low binding energy and poor affinity to the electrolyte.

**Fig. 5:**
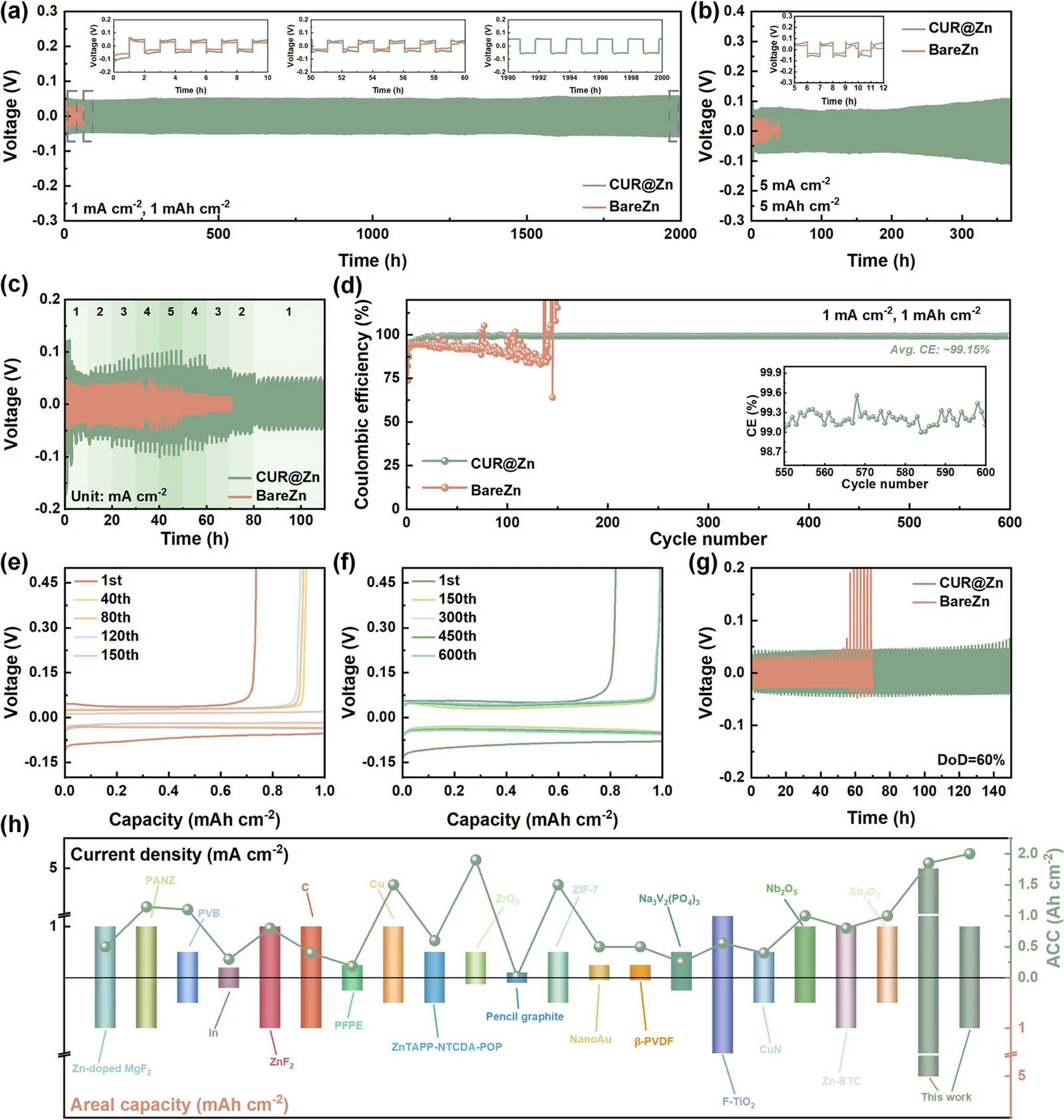
Voltage profiles of Zn||Zn symmetrical cells with different Zn foils at **a** 1 mA cm^−2^, 1 mAh cm^−2^, and **b** 5 mA cm^−2^, 5 mAh cm^−2^, insets show magnified V-t curves. **c** Rate performance of Zn||Zn cells. **d** CE curves of Zn||Cu cells at 1 mA cm^−2^, 1 mAh cm^−2^. Voltage profiles of Zn||Cu cells **e** without the curcumin layer, and **f** With the curcumin layer. **g** Voltage profiles of Zn||Zn cells at the depth of discharge of 60%. **h** Comparison of Zn||Zn symmetrical cell performance in published works employing the artificial protective layer

The durability of symmetrical cells was measured for both the low and the high current densities to confirm the improved performance for a wide range of operation conditions. As shown in Fig. 5a, without the curcumin protective layer, unstable voltage signals were observed after 50 h of operation, equivalent to only 26 cycles at 1 mA cm^−2^. In contrast, through the protection of the ultra-thin curcumin layer, cells can operate stably for more than 2000 h. This stark difference indicates the poor reversibility of bare Zn anodes in the Zn(OTf)_2_ electrolyte caused by the continuous corrosion, passivation, and dendrite growth. Meanwhile, when the current density is elevated, parasitic reactions will become increasingly more severe because of the boosted rate of reaction. Thus, the feasibility of adopting a higher current needs to be further confirmed, especially for the Zn(OTf)_2_ salts, which show no tolerance to high current and voltage. As shown in Fig. 5b, when the current density was set to 5 mA cm^−2^, the curcumin layer extended the lifetime of symmetrical cells from less than 10 h to about 400 h, successfully supplementing the potentials for the practical application. Moreover, the advancement of the stability of zinc anodes was also exhibited in cells with other working current densities and areal capacities (Fig. S30). A more comprehensive verification of the feasibility of stable operation under the broad range of current conditions was conducted by carrying out the rate performance evaluation. The current density rises first, then falls between 1 and 5 mA cm^−2^ with a step of 1 mA cm^−2^. For each current density, cells would operate for 5 cycles, then move forward. The final long-term operation was set at 1 mA cm^−2^. With the BareZn anode, the cell failed soon when the current density was ramped up to 4 mA cm^−2^. On the contrary, stable operation can be observed for all ranges of current densities when electrodes were replaced with the CUR@Zn (Fig. 5c). Meanwhile, with the continuous operation, the contact between the electrolyte and the curcumin protective layer became increasingly more solid and homogeneous. As a result, once the activation was achieved after the ramping stage, the overpotential was successfully reduced for the declining stage, and the final voltage variation became stable.

The failure of the zinc anode is directly caused by the zinc loss, and one primary factor influencing the loss rate of active zinc sources is the reversibility of zinc plating/stripping. Thus, the Coulombic efficiency (CE) was measured employing the Zn||Cu half batteries to assess the reversibility. Considering the fast corrosion rate and generation of passive products confirmed in the previous section, the utilization efficiency of zinc is anticipated to be non-ideal. Thus, the CE showed a decreasing trend after 30 cycles, and the half-cell became unstable in the following 20 cycles at 1 mA cm^−2^ (Fig. 5d). On the contrary, by coating the curcumin layer on both the zinc and copper foil, electrodes were shielded from the water-induced parasitic reactions, avoiding the zinc loss. At the same time, the boosted attractive force to Zn^2+^ facilitates the Zn^2+^ transportation kinetics for both the plating and stripping processes and relieves the issue of the dead zinc. As a result, the CE was improved up to 99.15% even after the stable operation of more than 600 cycles. Additionally, the CE elevation is also confirmed with other current densities (Fig. S31). The cell degradation can be more obvious in the charging/discharging profiles of half-cells. As indicated in Fig. 5e, f, a favorable initial cycle CE elevation from 73.64% to 82.06% was detected once the curcumin layer was applied. Meanwhile, the capacity decay never stopped for the BareZn symmetrical cells with a rapid rate of about 0.01 mAh cm^−2^ per 40 cycles. Contrarily, no degradation was detected with the CUR@Zn even for 150 cycles. The improved reversibility significantly improves the zinc utilization efficiency, encouraging potential for practical application with the limited zinc reservoir. Therefore, the depth of discharge (DoD) was elevated by replacing the thick zinc foils with copper covered with a limited amount of zinc, and the curcumin layer was coated on the copper foil, which was used as the substrate for the zinc-lean anode. When the DoD was advanced to 60%, a dramatic overpotential appeared after 50 h, indicating the depletion of zinc for the BareZn anode (Fig. 5g). Contrarily, the stable operation lasted for more than 150 h with the CUR@Zn, proving the high retention rate for the active zinc. From the perspective of the systematic energy density, even though the ex-situ protective layer introduces extra volume and mass, it paves the way to trim the excessive zinc supply. All-encompassing, the overall systematic energy density can be elevated to a level closer to industrialization (Fig. 5h, Table S2).

In summary, with the curcumin protective layer, the accumulative area capacity reached 2 Ah cm^−2^ with a CE of 99.15%. At the same time, with their extraordinary performance at the high current density, the favorable initial CE, and the promising stability exhibited with the bounded zinc supply, the curcumin protective layer brings the Zn(OTf)_2_-based AZIBs closer to the commercialization level. This advancement is outstanding compared to other ex-situ/in situ protective layer strategies.

The projection of stable zinc anodes in practical applications demands conjugation with suitable cathodes. Thus, NaV_3_O_8_·1.5H_2_O (NVO) was employed as the cathode to fabricate Zn||NVO full cells. Through the simple liquid–solid stirring method, Na^2+^ and H_2_O molecules were successfully co-inserted into V_2_O_5_, which can be confirmed through the peak shift in XRD results (Fig. S32). The final NVO is in the form of nanobelts with a width of less than 1 μm with a uniform distribution of Na^+^, which can be demonstrated through the SEM image and corresponding elemental mapping analysis (Fig. S33).

**Fig. 6:**
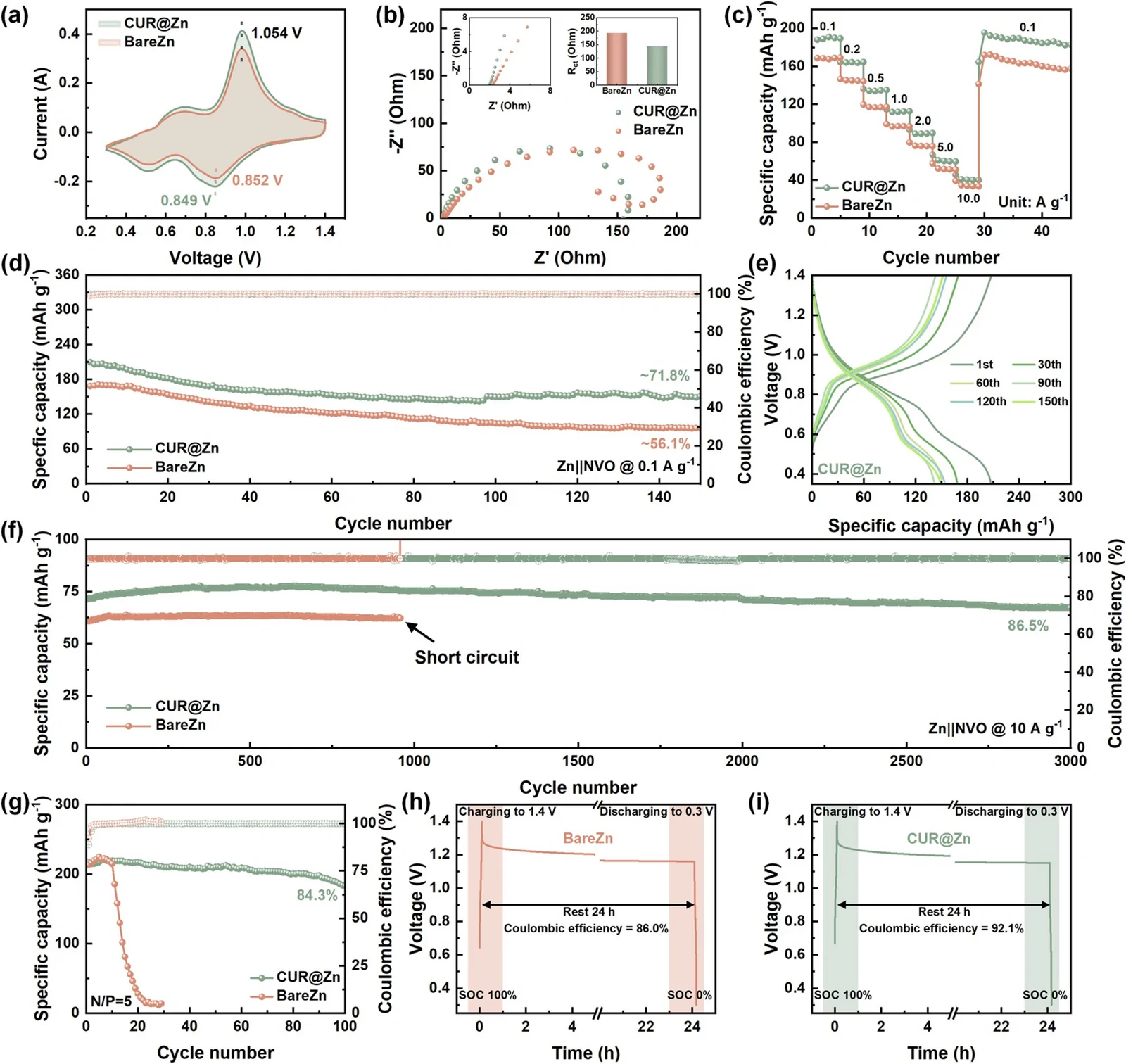
**a** CV curves of Zn||NVO batteries with a scan rate of 0.1 mV s^−1^. **b** Nyquist plots of Zn||NVO full cells with BareZn and CUR@Zn anodes, insets show x-intercepts and R_ct_. **c** Cycling performance of Zn||NVO batteries with varying specific current densities, showing the rate performance. **d** Cycling performance of Zn||NVO batteries at 0.1 A g^−1^, and **e** Corresponding charge–discharge curves of CUR@Zn||NVO battery. Cycling performance of Zn||NVO batteries **f** At 10 A g^−1^, and **g** With a N/P ratio of 5. Self-discharge curves of Zn||NVO cells with **h** BareZn anode and **i** CUR@Zn anode

The cyclic voltammetry (CV) method was first carried out for evaluating electrochemical reactions. As shown in Fig. 6a, two pairs of index peaks can be observed, matching the insertion and extraction of Zn^2+^. Similar peaks appeared for both full batteries with the BareZn anode and the CUR@Zn anode, so no undesired reactions happened with the introduction of the curcumin layer. Two oxidation peaks are located at the same voltage. On the contrary, one reduction peak shifted from 0.852 to 0.846 V with the curcumin layer. This proves a smaller voltage gap. Furthermore, at all these redox potentials, where reactions happened, the current densities were much higher, presenting decreased polarization in full cells. The facilitation of Zn^2+^ was also confirmed in the full battery by carrying out the EIS test. As shown in Fig. 6b, the Ohmic resistance is about 2.5 Ω, which is similar for both anodes. However, the charge transfer impedance (*R*_ct_) showed a dramatic decrease from 192.4 to 143.5 Ω with the casting of zincophilic curcumin layer. This boosting kinetics can be ascribed to the more forceful binding between the curcumin and Zn^2+^. As a result, the promotion of the anode interfacial kinetics can reduce the polarization, which is beneficial for improving the performance by presenting a higher practical capacity [51]. The rate performance was then evaluated by cycling full batteries at different current densities, which increased gradually from 0.1 to 10 A g^−1^, to verify the advanced kinetics. As shown in Fig. 6c, a high capacity of 195.6 mAh g^−1^ was exhibited for the CUR@Zn||NVO battery after the current density was recovered to 0.1 A g^−1^, surpassing the unshielded counterpart (172.0 mAh g^−1^). Thus, the capacities for the whole range of current densities are larger, and the operation is stable, indicating the favorable adaptability to working conditions. The stability was then evaluated at the specific current density of 0.1 A g^−1^. Considering the high capacity of the NVO cathode and the relatively small current, the operation time of one charging/discharging cycle is long, approximately 3 h. Because of the self-discharge and the dissolution of NVO in water, the capacity decay was much more obvious at the low current density. As a result, the degradation became more obvious at the low current density. With the BareZn anode, the capacity retention was only 56.1% after 150 cycles. On the contrary, for the CUR@Zn||NVO battery, after 60 cycles, the specific capacity was maintained at 150 mAh g^−1^ with negligible degradation (Fig. 6d, e). The overall capacity retention was up to 71.8% after 150 cycles. On the contrary, even though the capacity also reached stability after 90 cycles with the BareZn anode, only a capacity of about 90 mAh g^−1^ was maintained. (Fig. S34). Meanwhile, the capacity degradation for the first 15 cycles contributes most to the overall decay. For the BareZn anode, the capacities for the 1st, 15th, and 150th cycles were about 169, 160, and 94.8 mAh g^−1^, so the capacity decay for the first 15 cycles was about 5.3%, and for the following 135 cycles was about 38.6%. On the contrary, with the CUR@Zn anode, the capacities for the 1st, 15th, and 150th cycles were about 209.8 mAh g^−1^, 188 mAh g^−1^, and 150.6 mAh g^−1^, indicating the capacity decay of 10.4% for the first 15 cycles and of 17.8% for the following 135 cycles The efficacy of this organic layer was also assessed for the fast charging/discharging operation by setting the specific current density to 10 A g^−1^. As shown in Fig. 6f, after activation, the initial capacity reached 71.38 mAh g^−1^, which was higher compared to the value shown in the rate performance evaluation, experiencing the capacity degradation at low current densities. As a result, even after a prolonged cycling of 3000 cycles, the capacity of the CUR@Zn||NVO was still 67.28 mAh g^−1^, which was 86.5% of the maximum capacity. Contrarily, the BareZn||NVO battery failed in 1000 cycles. The stability at both low and high current densities is favorable compared to recent reported strategies using the artificial protective layer. Meanwhile, the performance at the high specific current density is especially superior (Table S3). The sharp contrast emphasizes the effectiveness of a more robust zinc anode on the full battery and confirms that the curcumin layer can be kept secure even under the high current density. To fulfill the commercial demand, the improved anode stability should be mapped onto a higher energy density. Therefore, the stability under the zinc-lean condition was also tested. The zinc foil was replaced with the copper foil with a limited amount of zinc. The zinc was electronically deposited by controlling the capacity according to the desired negative–positive (N/P) ratio. As shown in Fig. 6g, with the extremely low N/P ratio of 5, the discharge suddenly dropped after 10 cycles and reached 0 soon. This confirms the fast exhaustion of zinc without the protection of the curcumin layer. In sharp contrast, with the curcumin layer, zinc can be shielded from corrosion, and the improved reversibility relieves the issue of dead zinc. As a result, up to 84.3% of the maximum capacity can be retained after 100 cycles. The preservation of active materials was subsequently assessed by testing the self-discharge phenomenon. The Zn||NVO batteries were fully charged. After resting for 24 h, the batteries started to discharge. The capacity loss during the resting period was evaluated by calculating the ratio between the discharge capacity and the charge capacity. As shown in Fig. 6h, i, for the BareZn||NVO, only 86.0% of the charge capacity remained after 24 h, and the CE was increased to 92.1% by using the CUR@Zn anode, validating the successful suppression of self-discharge. Furthermore, to identify the generality of the proposed strategy, Cu_x_V_2_O_5_ (CuVO), which is another form of V_2_O_5_ modified by the divalent ion, and Polyaniline (PANi) cathode, which is the conductive polymer, were used as the cathode. As shown in Fig. S35, the CUR@Zn||CuVO battery exhibited the capacities of 135.72 mAh g^−1^ and 271.1 mAh g^−1^ with high capacity retentions of 70.7% after 1800 cycles, and 72.2% after 300 cycles were exhibited at current densities of 10 A g^−1^ and 1 A g^−1^, respectively. With the PANi cathode, the full battery reached the maximum capacity of 72.65 mAh g^−1^, and the capacity retention of 90.2% at 1 A g^−1^. The superior performance confirms the general efficacy of the curcumin layer in the comprehensive intercalation-type V_2_O_5_ cathode and the coordination-type PANi cathode.

In summary, this ultra-thin curcumin protective layer facilitates the transportation of Zn^2+^ and reinforces the interfacial stability. As a result, the zinc anode becomes robust enough for the practical application of the Zn(OTf)_2_-based system. Moreover, extraordinary performance when the zinc amount is reduced effectively elevates the energy density, paving the way for future industrialization and commercialization.

## Conclusion

In summary, the degradable curcumin was employed as an effective artificial layer to protect the zinc anode. The curcumin can be finely mixed with PVDF to form a purely liquid precursor to ensure the homogeneity and the robust adhesion of the protective layer. Meanwhile, the organic skeletons inhibited in this layer provide a favorable adhesion to the Zn(OTf)_2_ electrolyte, and polar functional groups on the curcumin act as anchor points of Zn^2+^ to ensure a facilitated transportation of charge carriers. As a result, this 2 µm artificial layer shields the zinc anode from water-induced parasitic reactions, and the uniform distribution of Zn^2+^ results in the flat and even deposition of zinc. Consequently, the CUR@Zn anode contributes to a superiorly durable operation of Zn||Zn symmetric cells for more than 2000 h at 1 mA cm^−2^. Moreover, the Coulombic efficiency reached 99.15% even after 600 cycles, and a high DoD of 60% was also achieved, confirming the advancement of the utilization efficiency of zinc. This optimized anode further demonstrated its efficacy in the practical scenario by exhibiting an astonishing capacity retention of higher than 86.5% in Zn||NVO full batteries even after 3000 cycles. Our findings fabricated a convenient, environmentally friendly, and scalable artificial layer by using the simple blade coating method to decorate the zinc anodes with curcumin. This method significantly improved the stability and reversibility of the zinc anode, providing a feasible and handy strategy to bring the Zn(OTf)_2_-based AZMBs to a level fulfilling the requirements of commercialization.

## Supplementary Information

Below is the link to the electronic supplementary material.

Supplementary file1 (DOCX 10446 KB)
