# Supplementary material for: Creation of an Artificial Layer for Boosting Zn2+ Mass Transfer and Anode Stability in Aqueous Zinc Metal Batteries
Source: Nanomicro Lett. 2026 Jan 15;18:121. doi: 10.1007/s40820-025-01973-0 (PMC12804491; doi:10.1007/s40820-025-01973-0)
Supplement: Supplementary file 1 — Supplementary file1 (DOCX 10446 KB) [file 40820_2025_1973_MOESM1_ESM.docx]

s

**Creation of an Artificial Layer for Boosting Zn^2+^ Mass Transfer and Anode Stability in Aqueous Zinc Metal Batteries**

Mingcong Tang^1^, Qun Liu^4^, Gang Liu^1^, Xiaohong Zou^1^, Kouer Zhang^1^, Zhenlu Yu^2^, Biao Zhang^2,^ *, Liang An^1,3,^ *

^1^ Department of Mechanical Engineering, The Hong Kong Polytechnic University, Hung Hom, Hong Kong SAR 999077, P. R. China

^2^ Department of Applied Physics, The Hong Kong Polytechnic University, Hung Hom, Hong Kong SAR 999077, P. R. China

^3^ Research Institute for Smart Energy, The Hong Kong Polytechnic University, Hung Hom, Kowloon, Hong Kong SAR 999077, P. R. China

^4^ College of Energy Materials and Chemistry, College of Chemistry and Chemical Engineering, Inner Mongolia University, Hohhot 010021, P. R. China

*Corresponding authors. E-mail: [biao.ap.zhang@polyu.edu.hk](mailto:biao.ap.zhang@polyu.edu.hk) (Biao Zhang); [liang.an@polyu.edu.hk](mailto:liang.an@polyu.edu.hk) (Liang An)

**S1 Experimental Section**

**S1.1 Assembly of Coin Cell and Electrochemical Test**

Coin-type 2032 battery cases were employed for all battery tests, with the Whatman GF/D glass fiber as the separator. 1.0 M Zn(OTf)_2_ aqueous solution was used as the electrolyte. For asymmetric cells, 0.05 mm Cu foil was used as the cathode. LSV, I-t, EIS, Tafel, CV, and differential capacitance-potential curves were obtained with an electrochemical workstation (BioLogic, VMP-300). The cycling performance of symmetric cells and full cells, rate performance, CE, and nucleation overpotential were evaluated using a battery testing system (Neware CT-4008) at ambient temperature. For all full cell tests, the cells were cycled at 0.1 A g^-1^ for 10 cycles to activate the battery. The ion transference number was measured using the Bruce-Vincent method. The chronoamperometry(CA) test was conducted with a symmetric cell at a constant bias of 20 mV, which is denoted as V. The currents at the beginning and reaching the stability are denoted as I_0_ and I_s_, respectively. The EIS measurements were conducted before and after the CA test, and charge transfer resistances are R_0_ and R_s_, respectively. Then, the transference number can be calculated using the following equation.

|  | $t_{{Zn}^{2+}}=\frac{I_{s}(V-I_{0}R_{0})}{I_{0}(V-I_{s}R_{s})}$ |  |
| --- | --- | --- |

The activation energy was obtained through the modified Arrhenius equation. The EIS of symmetric cells was measured at different temperatures ranging from 25 to 65 ℃ with a constant increasing gradient of 10. For each temperature, the cell would be stabilized for 30 minutes. Then the activation energy would be calculated via the following equation.

|  | $\frac{1}{R_{ct}}=Aexp(-\frac{E_{a}}{RT})$ |  |
| --- | --- | --- |

where R_ct_ is the charge transfer resistance, A is the Arrhenius factor, R is the universal gas constant, T is the Kelvin temperature, and E_a_ is the activation energy. Among them, A and R are constants. Once the relationship between -ln(R_ct_) and 1000/T is obtained, the activation energy can be evaluated from the slope.

The electric double layer capacitance (EDLC) value (C) was obtained by conducting CV tests of Zn||Zn symmetric cells in the range from -15 mV to 15 mV. The scanning rates changed from 8 mV s^-1^ to 16 mV s^-1^ with an increasing gradient of 2 mV s^-1^. The EDCL value was calculated with the following equation.

$$C=\frac{i_{C}}{v}$$

where ic is the average between the upper and lower current densities when the voltage is 0 V vs. Zn/Zn^2+^. The v is the scan rate.

**S1.2 Material characterization**

The morphology of the zinc foils after corrosion and cycling was observed using the scanning electron microscope (SEM, Tescan VEGA3). The thickness and morphology of the curcumin layer, and the morphology of NVO and curcumin powders, are observed with the field emission scanning electron microscope (FESEM, Tescan MIRA), and the energy dispersive X-ray spectroscopy (EDX) was carried out with the same equipment. Raman shift spectroscopy was conducted using Witec alpha 300R, and the laser source was set to 532 nm. The crystalline information was determined using the X-ray diffraction (XRD, Rigaku SmartLab 9kW-Advance) with Cu *K*_β_ radiation at 45 kV. X-ray photoelectron spectroscopy (XPS, Thermo Fisher Scientific Nexsa) was employed to study the surface chemistry of the curcumin powder and curcumin layer. The in-situ observation of deposition was carried out using the optical microscope. The surface roughness was tested with the 3D laser scanning microscope (KEYENCE VK-X200). Contact angles were measured with the optical tensiometer (Biolin Scientific Theta) to determine the adhesion between electrodes and electrolytes.

A conical indenter was employed to evaluate the robustness of the layer. Because the thickness of the layer is thin, the maximum load was adjusted to ensure that the depth was falling in the range of 2 μm. The final maximum load was decided to be 5 mN. In this equation, A is the carved area, and S is the stiffness, which is the slope of the linear part of the unloading curve.

|  | $E=\frac{\sqrt{\pi}}{2}\frac{S}{\sqrt{A}}$ |  |
| --- | --- | --- |

**S1.3 Computational method**

For the calculation of the electrostatic mapping and Gibbs free energy change during the desolvation, the Gaussian 16 software was employed, also with the Perdew-Burke-Ernzerhof0 (PBE0) functional [S1]. The geometry optimization and frequency calculation were first carried out with the def2-SVP basis set to determine the optimal geometry of each compound [S2]. Followingly, the larger basis set, which is the def2-TZVP basis set, was used for the single-point energy calculations. The weak interaction was then corrected to improve the accuracy of calculation with the DFT-D3 dispersion correction with the BJ-damping [S3]. Specifically, for the Gibbs free energy change, the SMD implicit solvation model was utilized to take the solvation effect into account [S4]. Finally, the single point energy of each compound was added to terms that experienced the free energy correction before to obatins the Gibbs free energy. After obtaining each Gibbs free energy, the free energy change of each step during the desolvation process can be calculated with the following equation.

$$\Delta G=G_{product}-G_{reactant}$$

Adsorption energies and binding energies were calculated based on the DFT theory. All DFT calculations were carried out with the Vienna ab initio simulation package (VASP) [S5-S7]. The Perdew-Burke-Ernzerhof (PBE) functional with the generalized gradient approximation was utilized to describe the electronic exchange and correlation. [S8]. Then, the projector augmented-wave (PAW) method was used to treat the interaction between the valence electrons and ionic cores [S9]. Meanwhile, for the K-points on a Monkhorst-Pack grid, a value of 2*1*1 was set along with a cut-off voltage of 500 eV. A small SIGMA of 0.05 was adopted for the Gaussian smearing scheme. All of these DFT calculations were performed until the goal of minimizing forces was achieved with a convergence criterion of 0.02 eV Å^-1^. The DFT-D3 method proposed in the previous publication was used for the vdW-dispersion energy correction [S10]. The binding energy was calculated with the following equation.

$$E_{binding}=E_{A}+E_{B}-E_{AB}$$

For adsortion energies, Zn(101), Zn(100), and Zn(002) planes were selected as the exposed surface to calculate. Based on the adsorption configuration of Zn on these three planes, the curcumin is then adsorbed. Finally, the adsorption energies for these configurations were calculated with the following equation.

$$E_{ads}=E_{*Comp}-E_{*}-E_{comp}$$

In this equation, $E_{*Comp}$ stands for the energy of the material with the adsorbed molecule; $E_{*}$ is the energy of the material without the adsorbed molecule; $E_{comp}$ is the energy of the molecule under vacuum.

**S1.4 Phase-field model**

The mechanism of the solid-liquid interface change, namely dendrite growth on the electrode surface and the change in electrolyte concentration, is described by means of COMSOL Multiphysics 6.2. The geometric model is simplified and set as a two-dimensional rectangle of 6 μm*6 μm. The top gives a fixed potential and concentration through the Dirichlet boundary condition, and the remaining boundaries are set as no flux. The initial conditions present three fixed initial nucleation sites at the bottom. The control equation is described as follows.

**Phase-field** **equation:** The growth of zinc dendrites in the Phase field equation is described in the phase field method as the microstructure characteristics represented by continuous variables, that is, different phases are represented by an order parameter ($\xi$).When $\xi$=0 for the solid electrode, $\xi$=1 for the liquid electrolyte. The continuous evolution process of this variable is facilitated by the interfacial free energy and coupled with the influence of the change in the electric potential field.

$$\frac{\partial\xi}{\partial t}=-L_{\sigma}\left( g^{'}\left( \xi\right)-\kappa\nabla^{2}\xi\right)-L_{\eta}h^{'}\left( \xi\right)\left\{ \exp\left[ \frac{\left( 1-\alpha\right)nF\eta_{a}}{RT} \right]+\tilde{c}_{+}\exp\left[ \frac{-\alpha nF\eta_{a}}{RT} \right] \right\}$$

Where $L_{\sigma}$ is the interfacial mobility, $L_{\eta}$ is the reaction rate constant. $g^{'}\left( \xi\right)=W\xi^{2}{(1-\xi)}^{2}$ is a double-well free energy function. $h^{'}\left( \xi\right)=\xi^{3}(6\xi^{2}-15\xi+10)$ is an interpolating function for the electrode–electrolyte interface. $\kappa=\kappa_{0}\left( 1+\delta\cos\left( \lambda\theta\right) \right)$ is the anisotropy surface energy for the depositing morphology, where $\kappa_{0}$ is a constant, $\theta$ is the angle between the normal vector of the interface and the reference axis, $\delta$ is the strength of surface energy anisotropy, and $\lambda$ is a mode number of the anisotropy. $\alpha$ is the transfer coefficient, n is the electron transfer number, *F* is the Faraday's constant, $\eta_{a}$ is the overpotential, *R* and *T* are the gas constant and the temperature, respectively, $\tilde{c}_{+}$ is the zincate ion concentration, and the initial electrolyte concentration, respectively.

**Concentration field equation:** The variation of ion concentration is controlled by diffusion, migration and electrochemical reaction consumption in the Nernste-Planck equation.

$$\frac{\partial\tilde{c}_{+}}{\partial t}\nabla\cdot\left[ \frac{D^{eff}\nabla\tilde{c}_{+}}{RT}nF\nabla\phi\right]-\frac{c_{s}}{c_{0}}\frac{\partial\xi}{\partial t}$$

$$D^{eff}=D^{e}h\left( \xi\right)+D^{S}(1-h\left( \xi\right))$$

Where $c_{s}$ and $c_{0}$ are the site density and the initial electrolyte concentration, respectively. $D^{eff}$ is the effective diffusion coefficient, which is defined by the diffusion coefficient of the metal cation in the electrode ($D^{e}$) and in the electrolyte ($D^{S}$).

**Electric-field equation:** Control the electric field based on the conservation of charge.

$$\frac{\partial\phi}{\partial t}+\nabla\cdot\sigma^{eff}\nabla\left( \phi\right)=\frac{\partial\xi}{\partial t}$$

$$\sigma^{eff}=\sigma^{e}h\left( \xi\right)+\sigma^{S}(1-h(\xi))$$

Where $\sigma^{e}$ and $\sigma^{S}$ are the conductivities of the electrode and electrolyte solution, respectively.

**S2 Supplementary Figures**

**
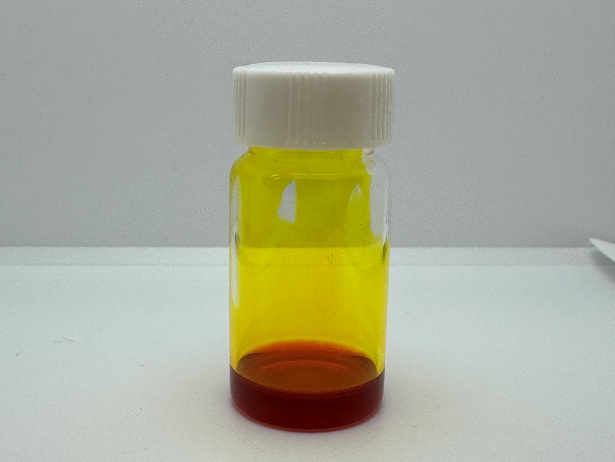
**

**Fig. S1** Digital image of the curcumin solution


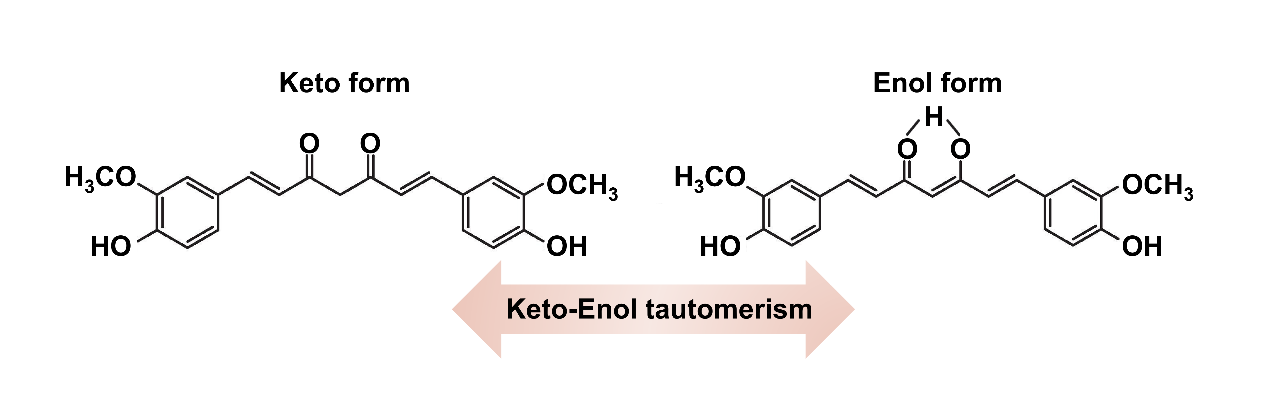


**Fig. S2** Schematic demonstration of the Keto-Enol tautomerism


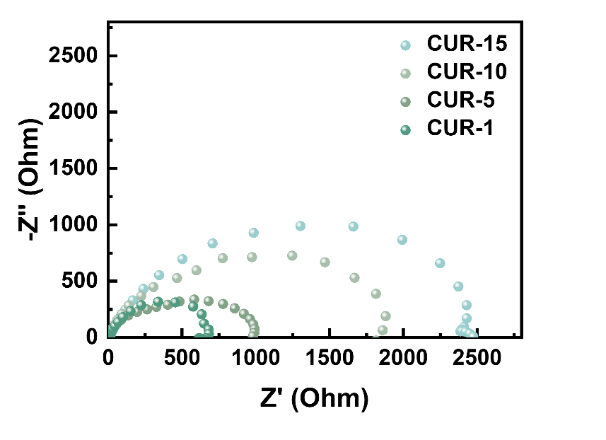


**Fig. S3** Nyquist plots of Zn||Zn symmetrical cells with zinc electrodes coated with different thicknesses of protective layers


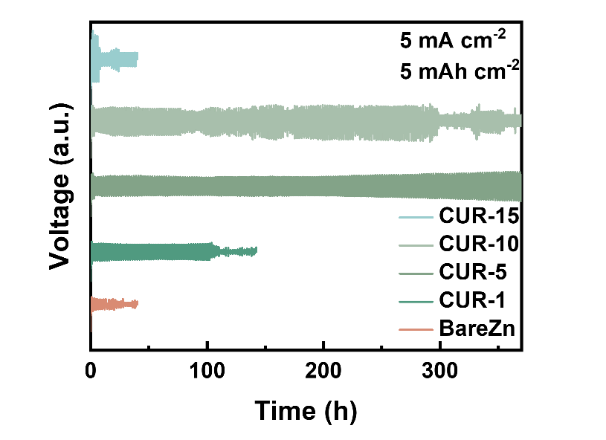


**Fig. S4** Voltage profiles of Zn||Zn symmetrical cells with different zinc anodes


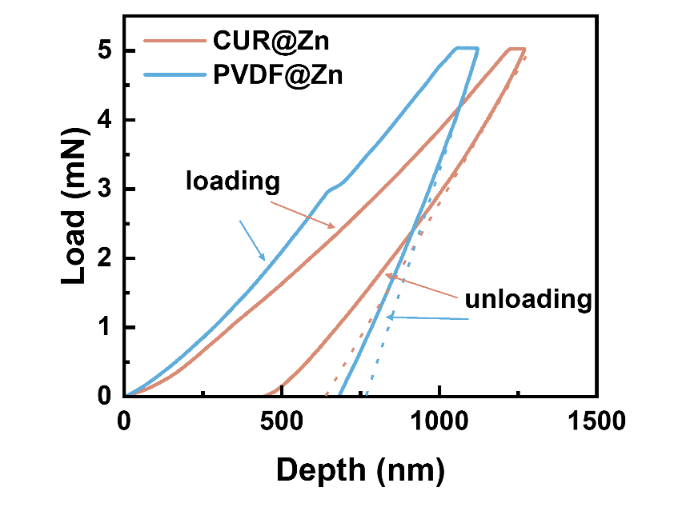


**Fig. S5** Load-displacement curves of the PVDF@Zn and CUR@Zn

**
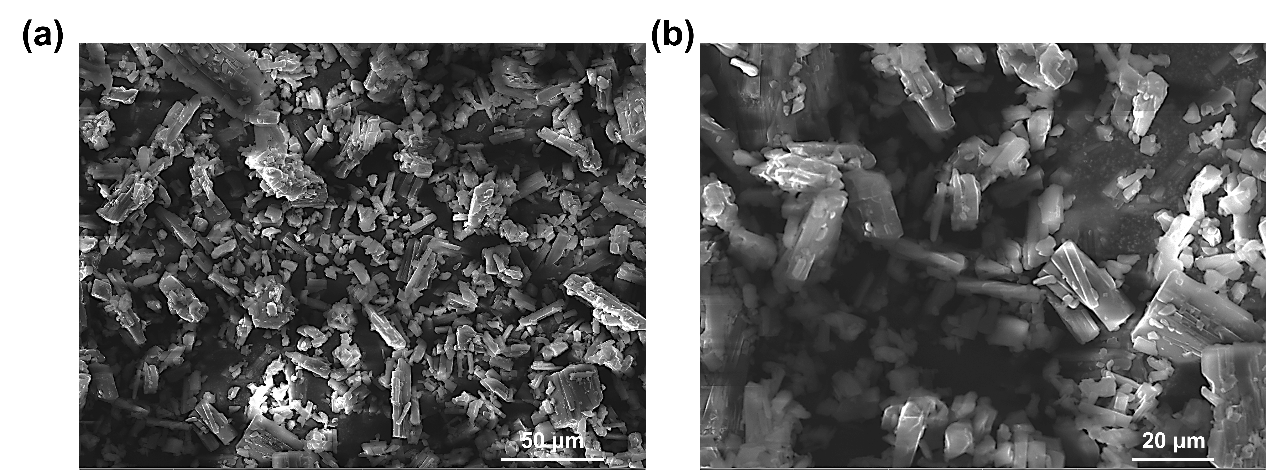
**

**Fig. S6** SEM images of the curcumin powder with the magnification of **a** 1000 times, and **b** 2000 times

**
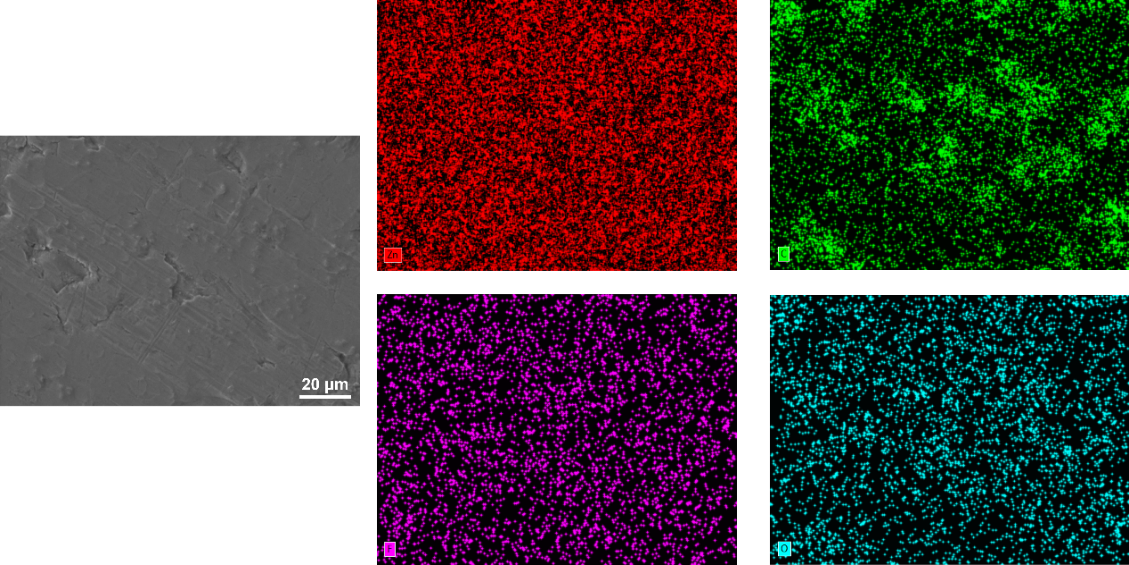
**

**Fig. S7** SEM image of the CUR@Zn foil with 2000x magnification, and the corresponding elemental mapping

**
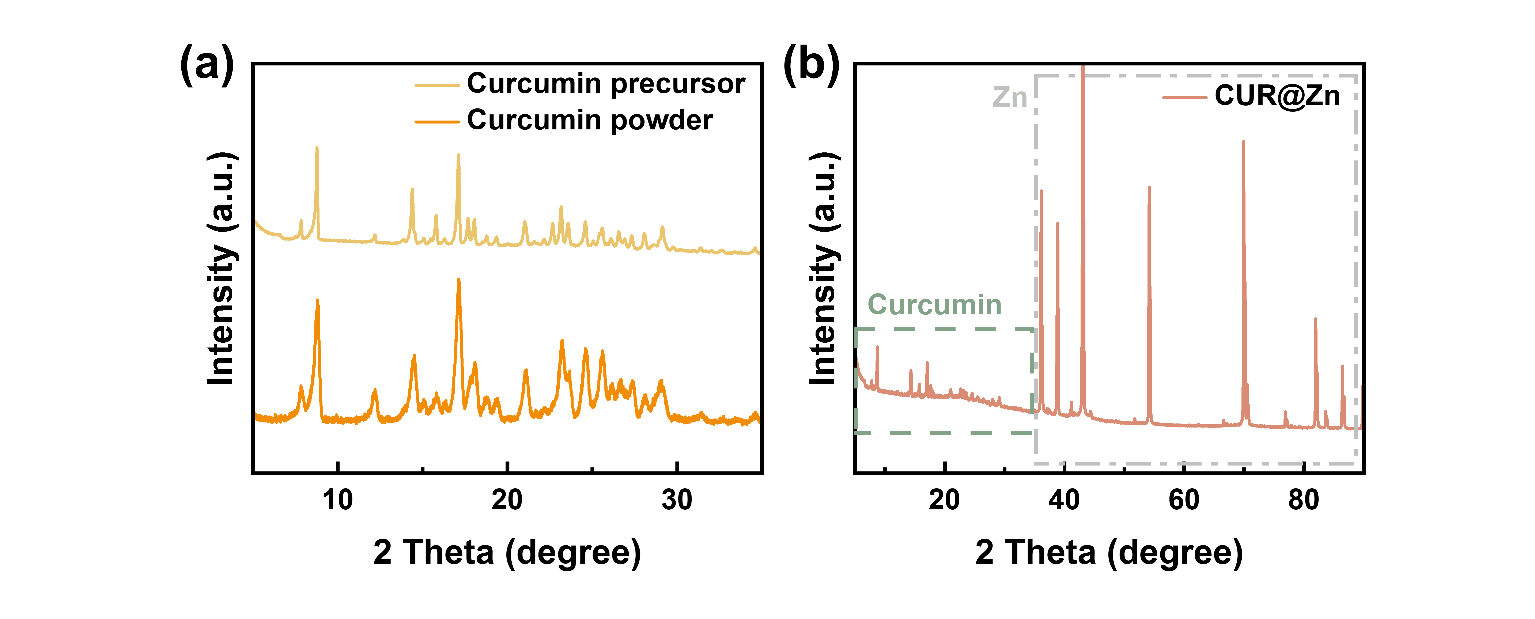
**

**Fig. S8** XRD patterns of **a** curcumin powder and curcumin layer precursor, and **b** curcumin layer-covered zinc foil


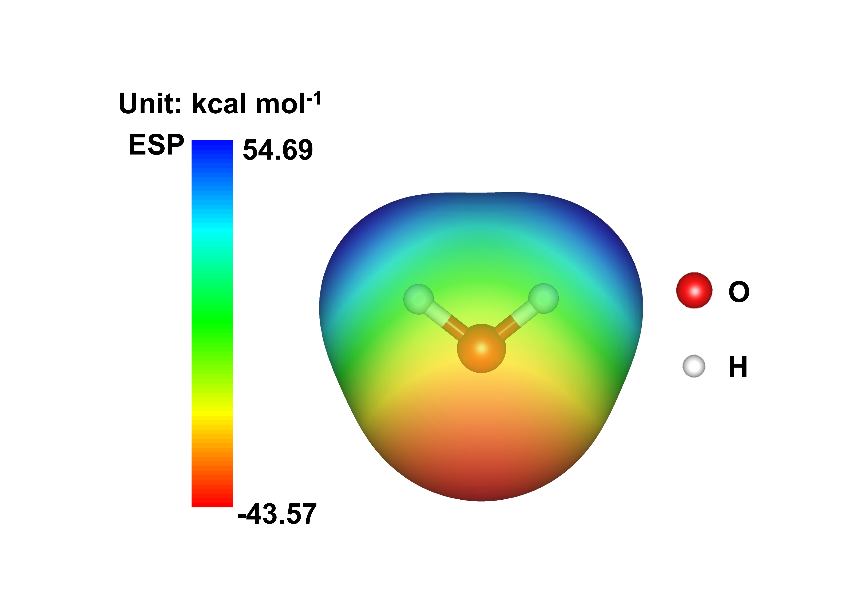


**Fig. S9** Electrostatic mapping of the water molecule


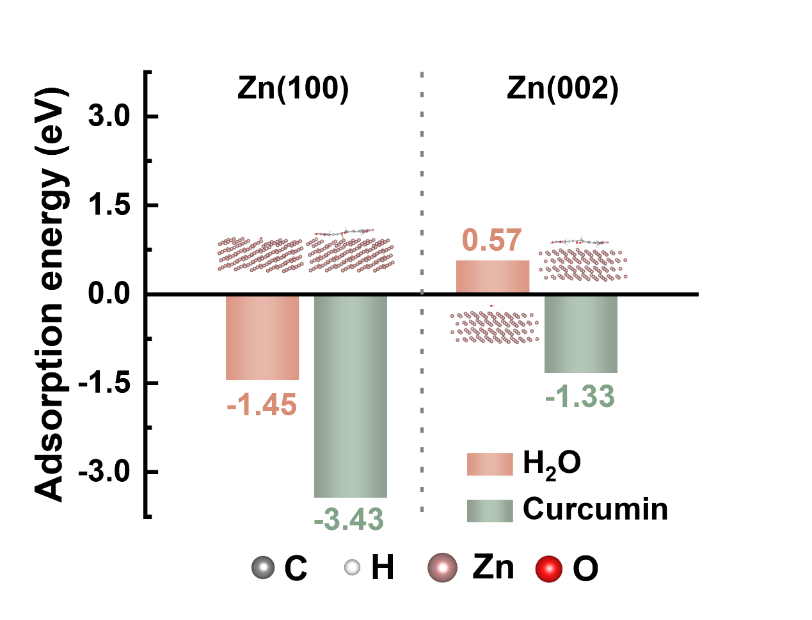


**Fig. S10** Adsorption energies of the H_2_O molecule and the curcumin molecule on Zn(100) and Zn(002) planes


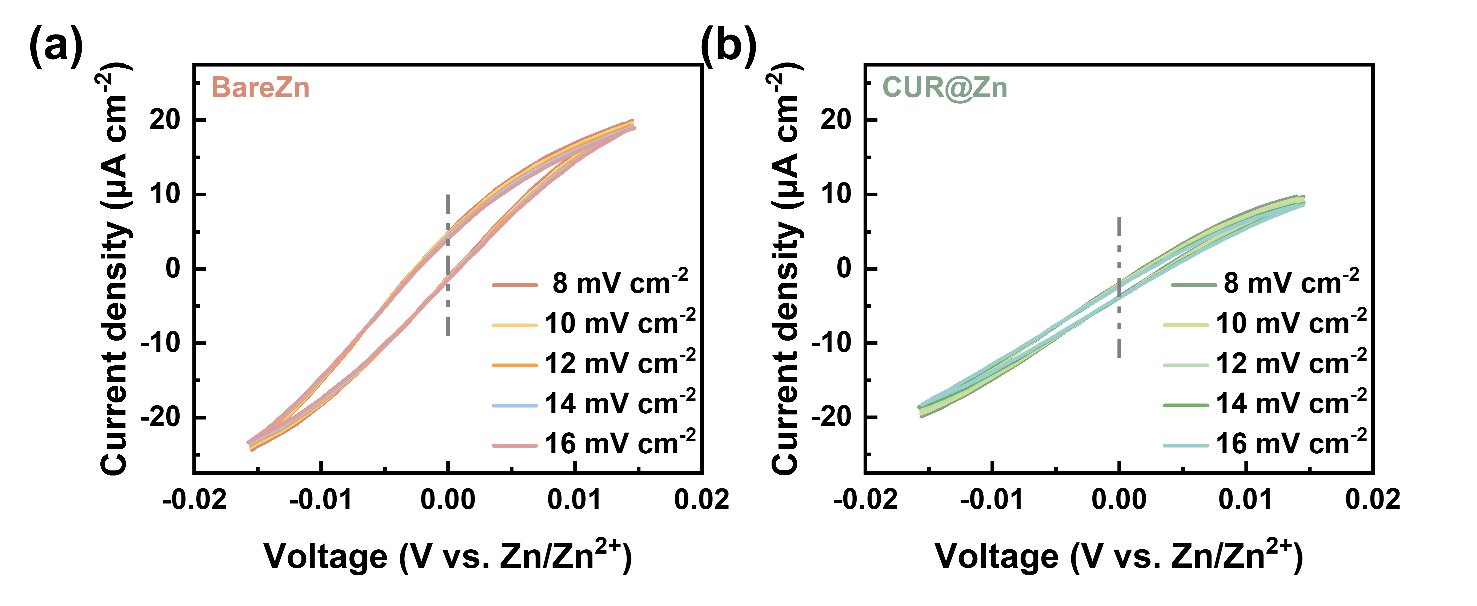


**Fig. S11** CV curves of Zn||Zn symmetrical cells at various scan rates from 8 mV s^-1^ to 16 mV s^-1^, with **a** BareZn anode, and **b** CUR@Zn anode


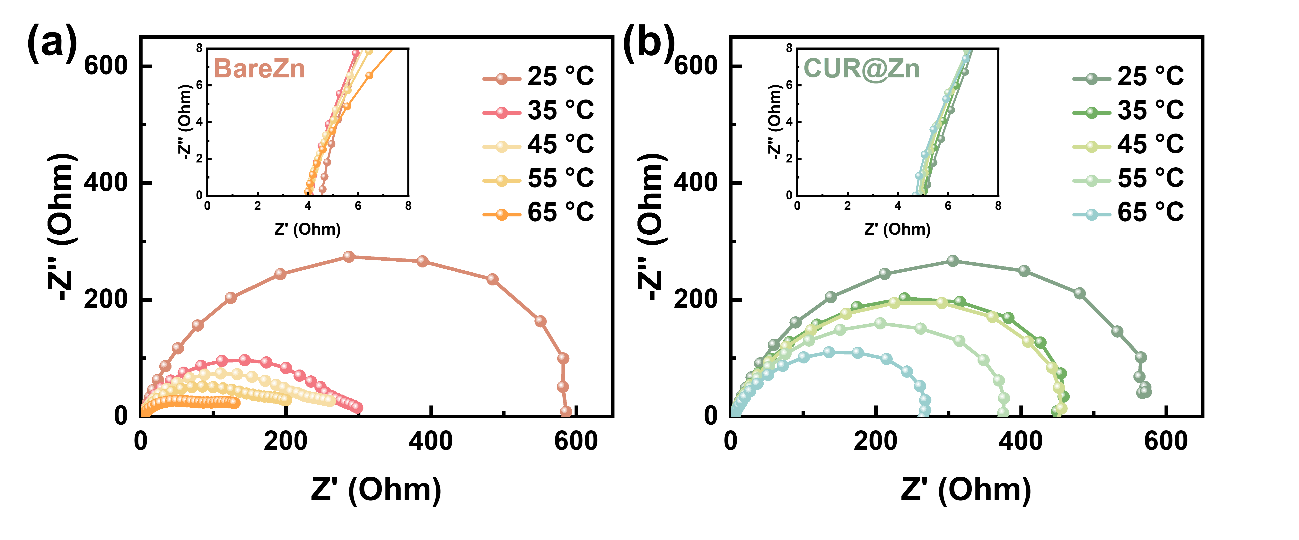


**Fig. S12** Nyquist plots at different temperatures of Zn||Zn symmetrical cells with **a** BareZn anode and **b** CUR@Zn anode


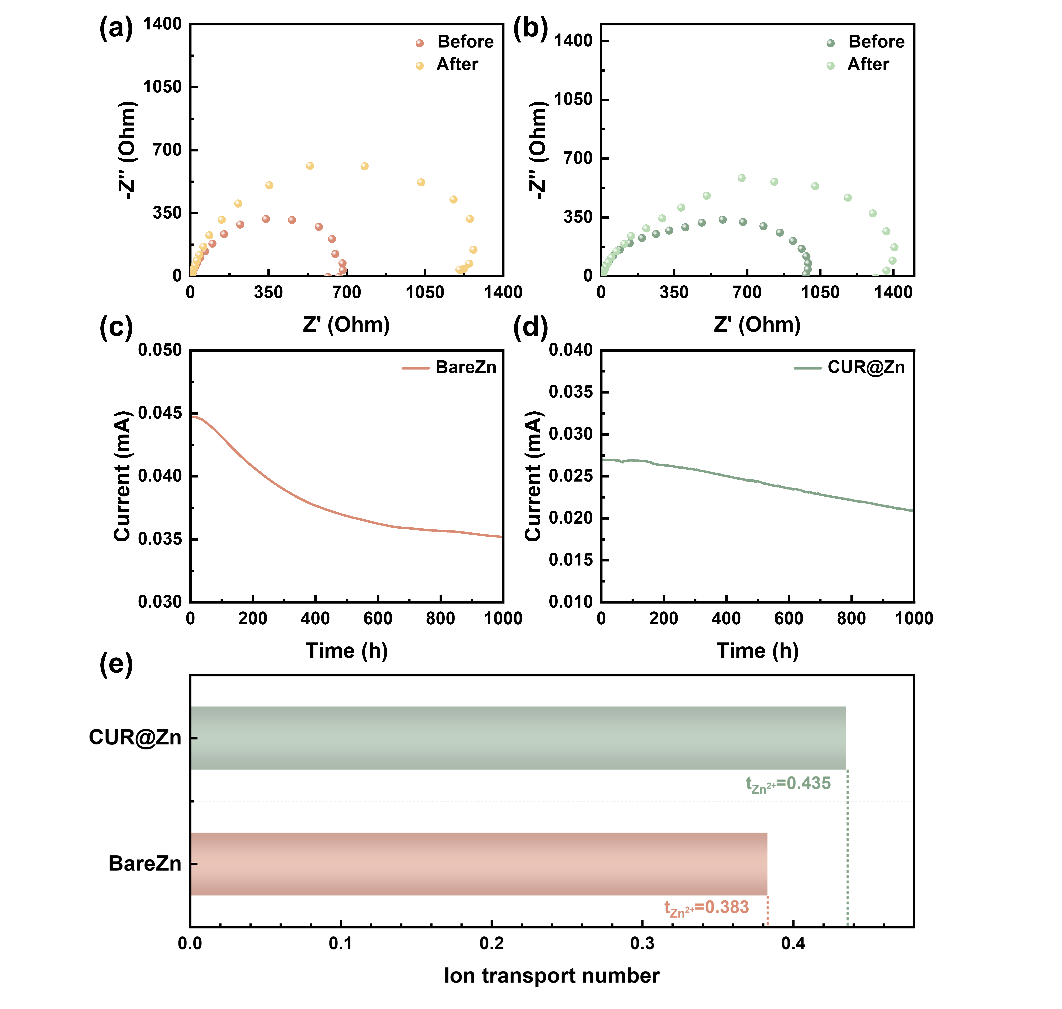


**Fig. S13** EIS curves of cells with **a** BareZn anode and **b** CUR@Zn anode. The associated i-t curve of cells with **c** BareZn anode and **d** CUR@Zn anode. **e** The calculated Zn^2+^ transference number


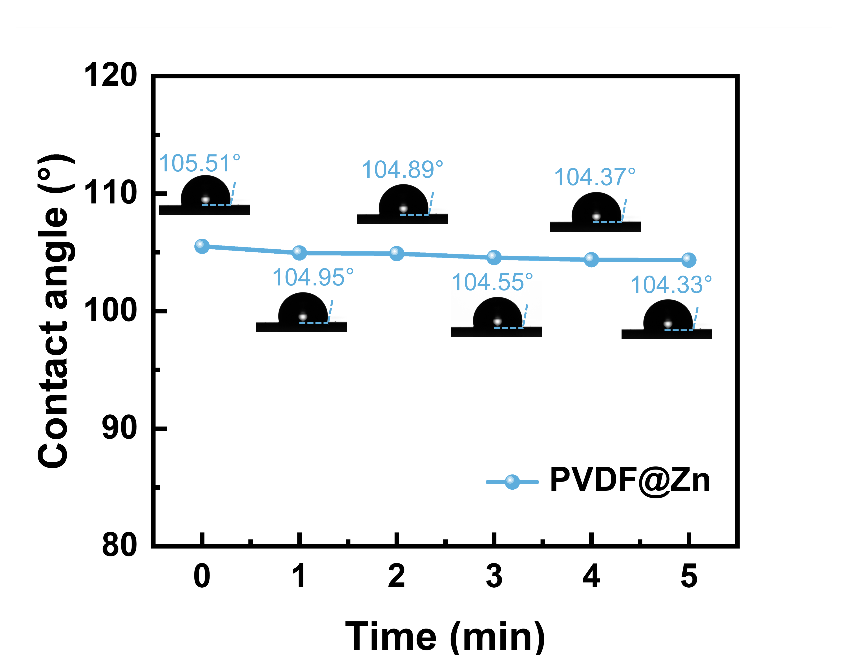


**Fig. S14** The contact angle between the electrolyte and the PDVF@Zn anode

**
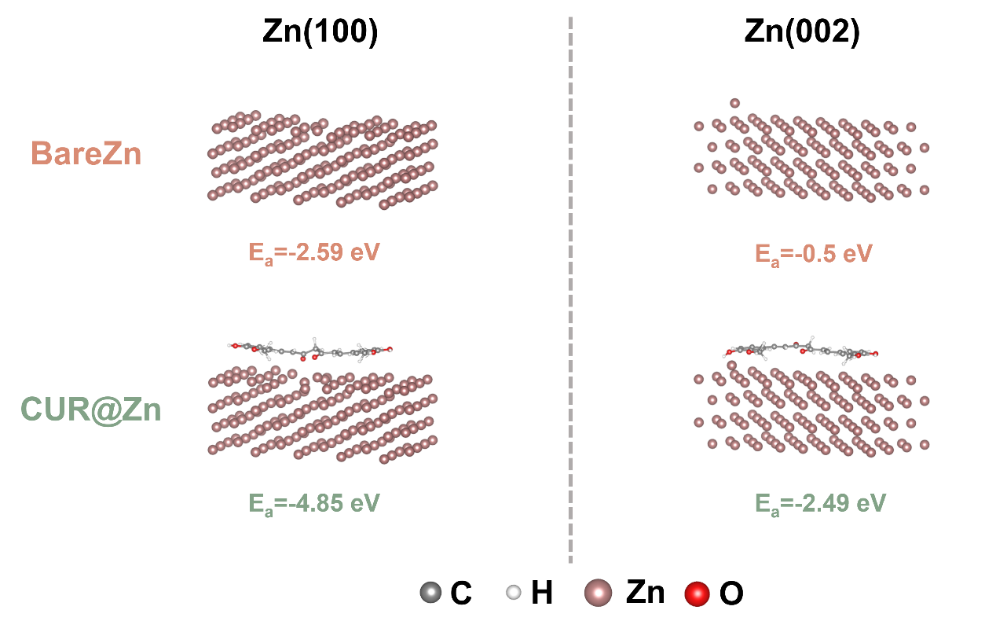
**

**Fig. S15** Adsorption energy of Zn^2+^ on the Zn(100) and Zn(002) planes of the BareZn and CUR@Zn anodes

8
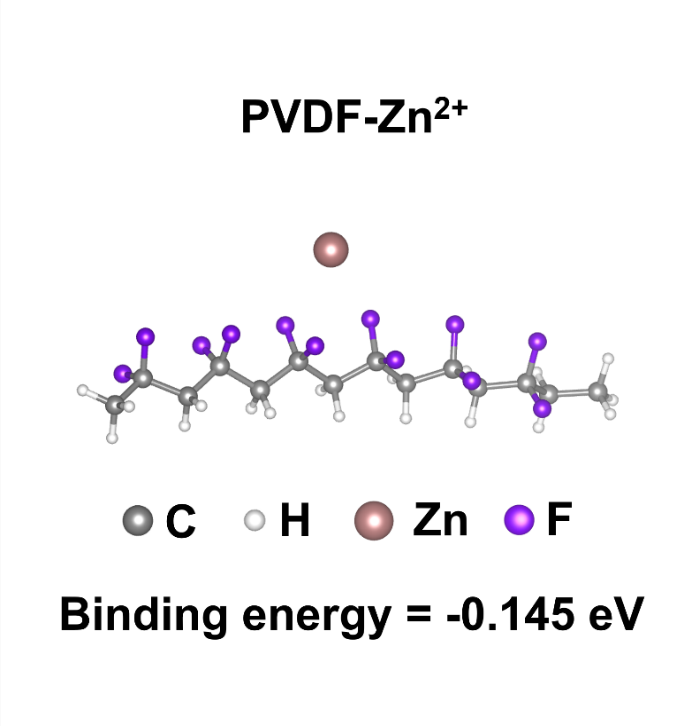


**Fig. S16** The binding energy between one section of PVDF and Zn^2+^


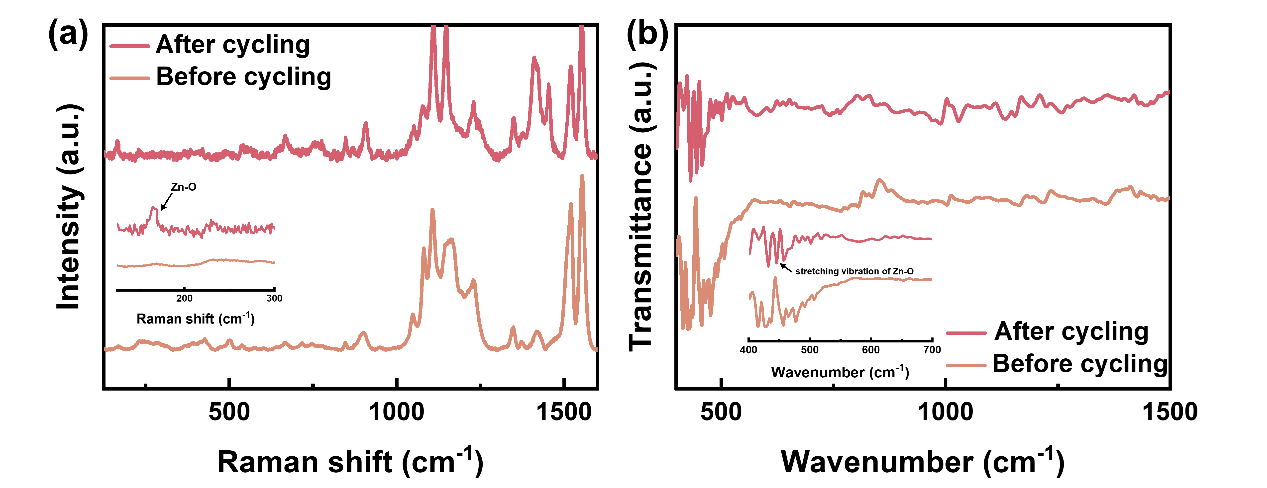


**Fig. S17** **a** Raman spectrum, and **b** FTIR spectrum of CUR@Zn foils before and after cycling for 30 cycles, insets show the new peak


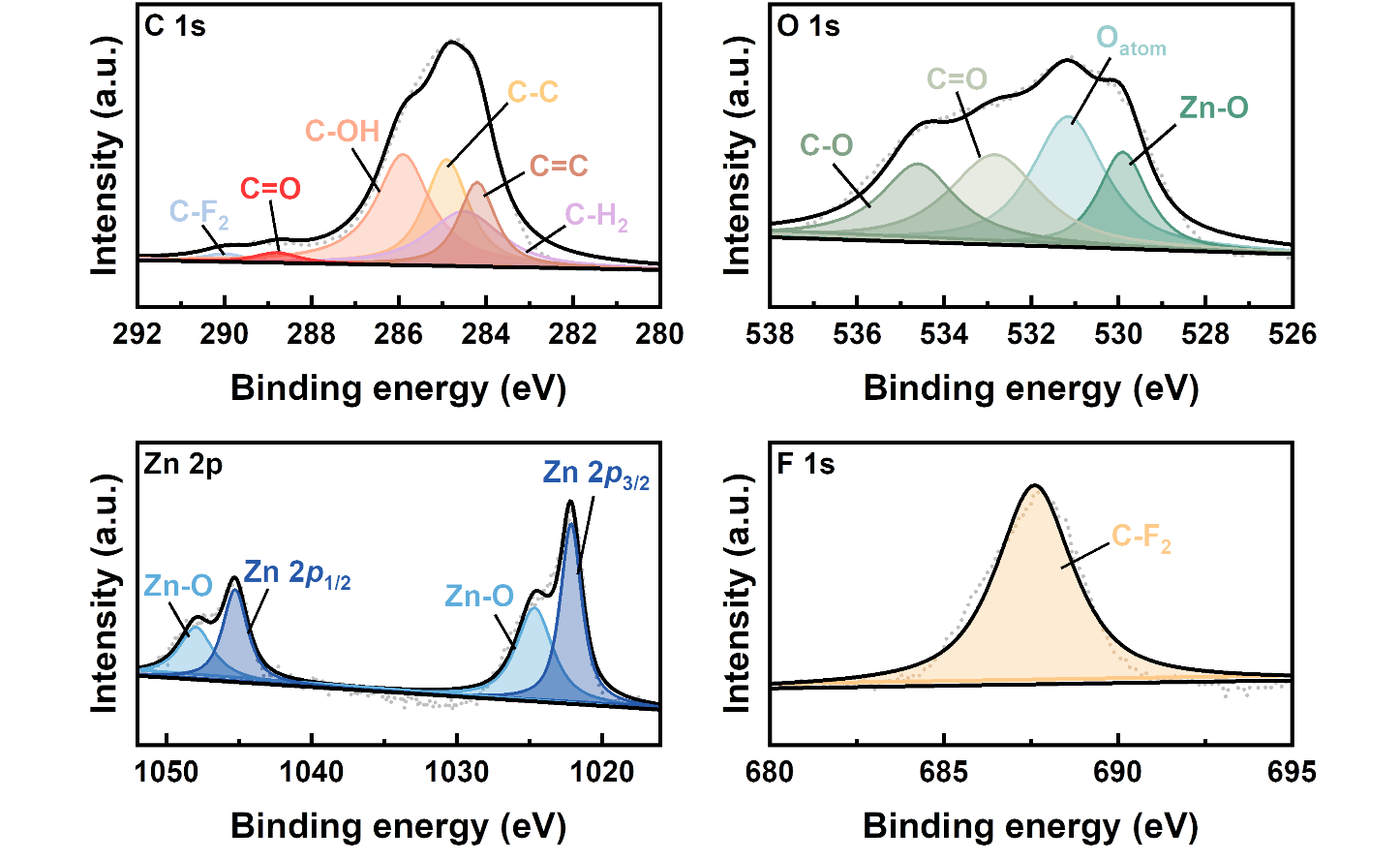


**Fig. S18** The high-resolution XPS C 1s, O 1s, Zn 2p, and F 1s spectra of the curcumin layer after cycling


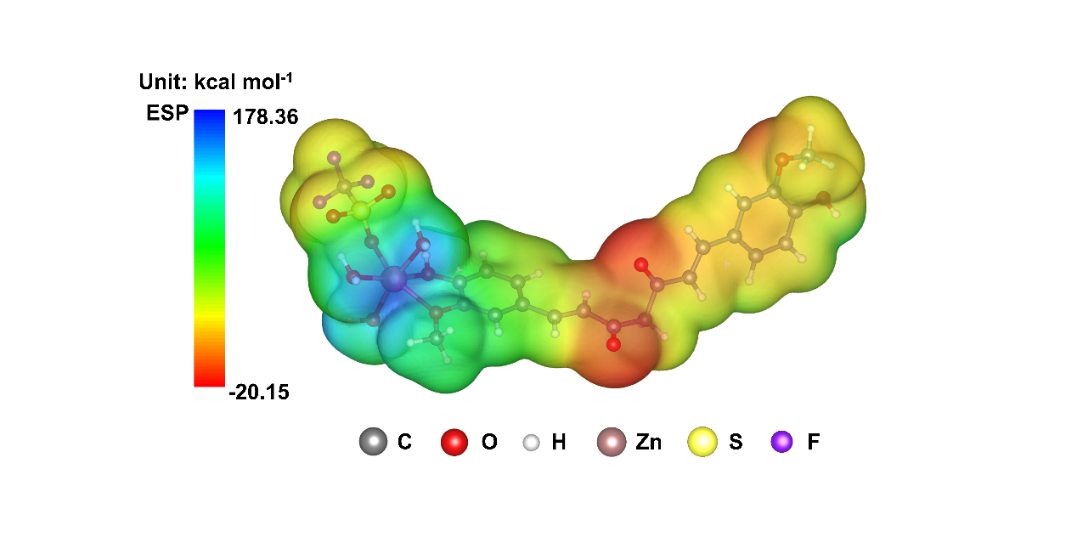


**Fig. S19** Electrostatic mapping of Curcumin-Zn^2+^-4H_2_O-OTf^-^, with Zn^2+^ attached on the -OH group


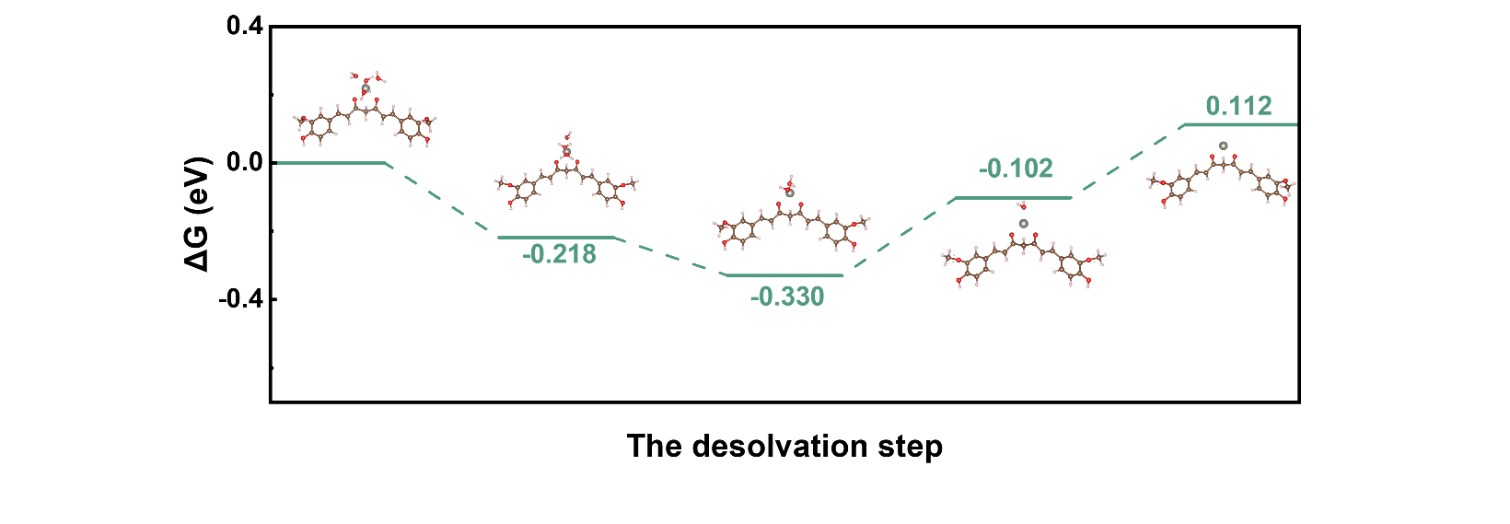


**Fig. S20** Profile of Gibbs free energy change for each step of the desolvation process with the curcumin participating in the solvation structure


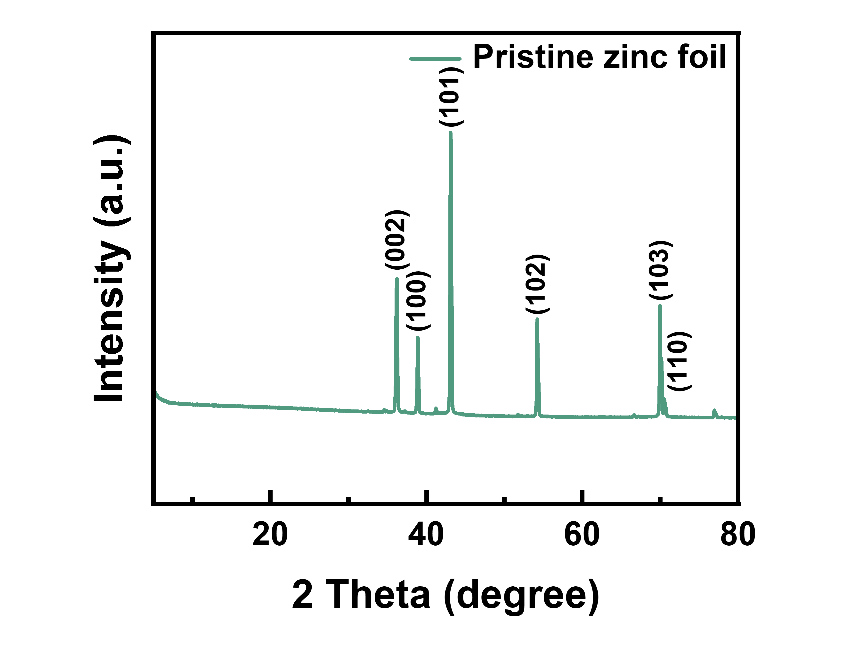


**Fig. S21** XRD pattern of the pristine zinc foil


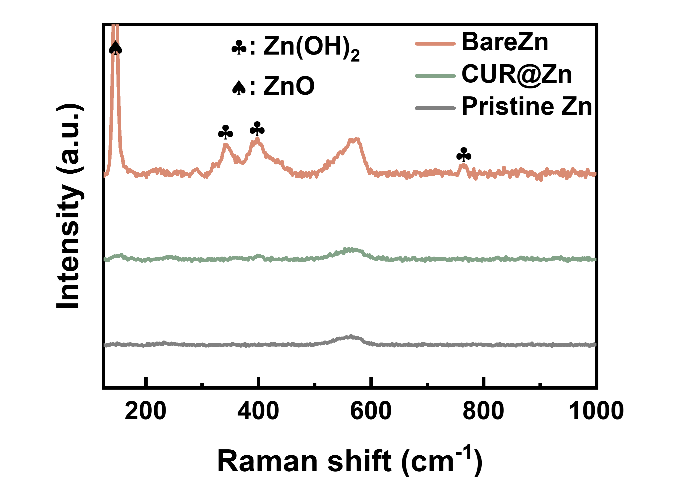


**Fig. S22** Raman spectra of different anodes soaked in the electrolyte for 100 h


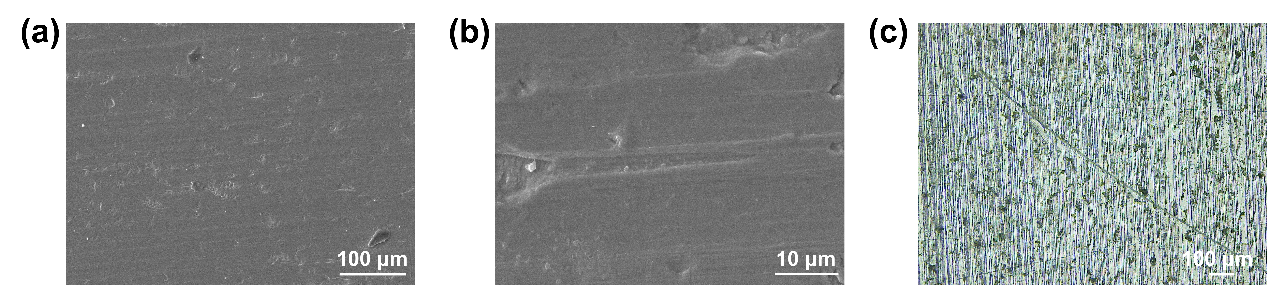


**Fig. S23** SEM images of the pristine zinc foil with the magnification of **a** 500 times, and **b** 5000 times. **c** Optical microscopic image of the pristine zinc foil


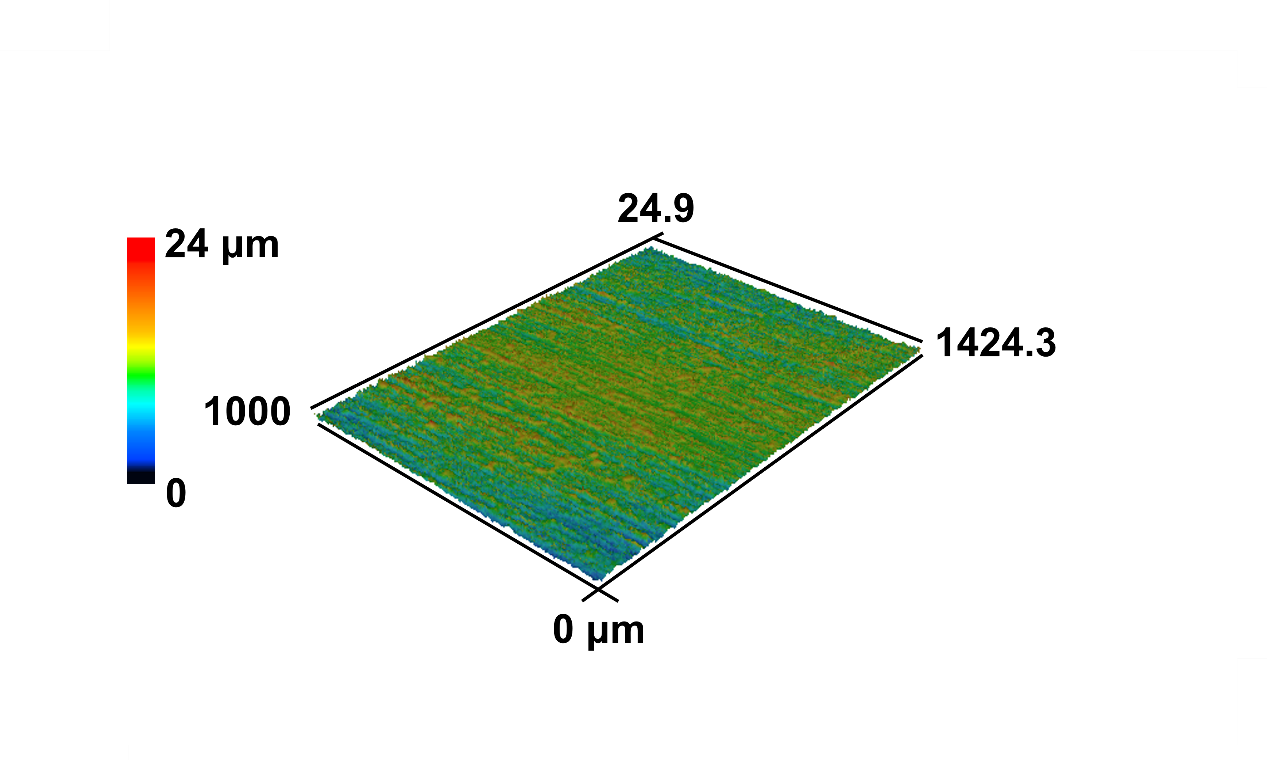


**Fig. S24** 3D topology obtained by CLSM analysis of the pristine zinc foil


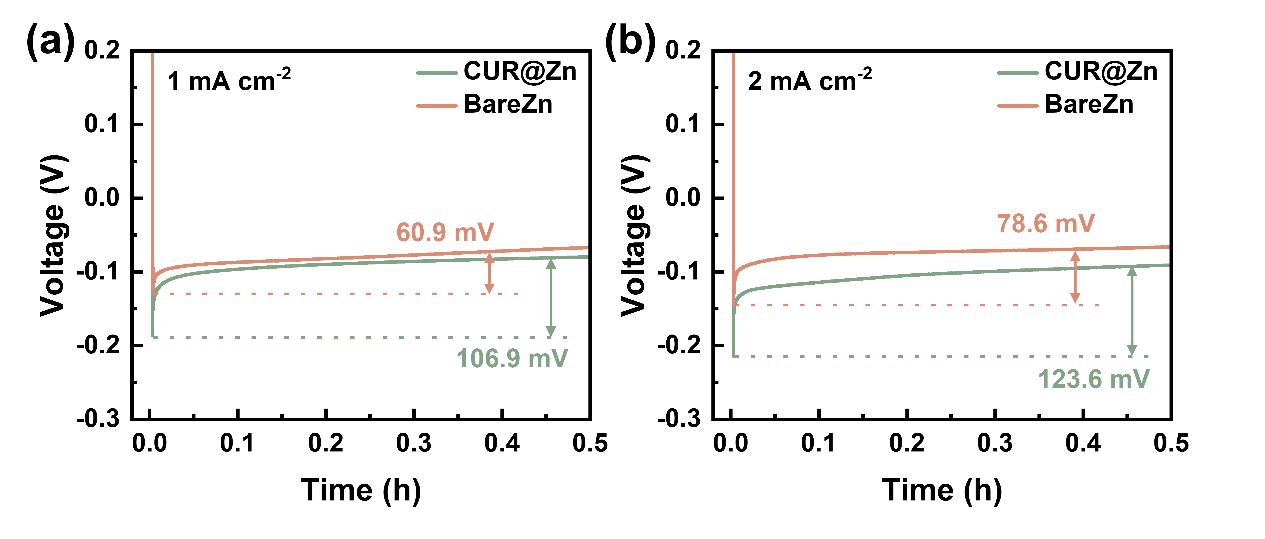


**Fig. S25** The initial nucleation overpotential using different anodes in Zn||Cu cells at the current density of **a** 1 mA cm^-2^, and **b** 2 mA cm^-2^


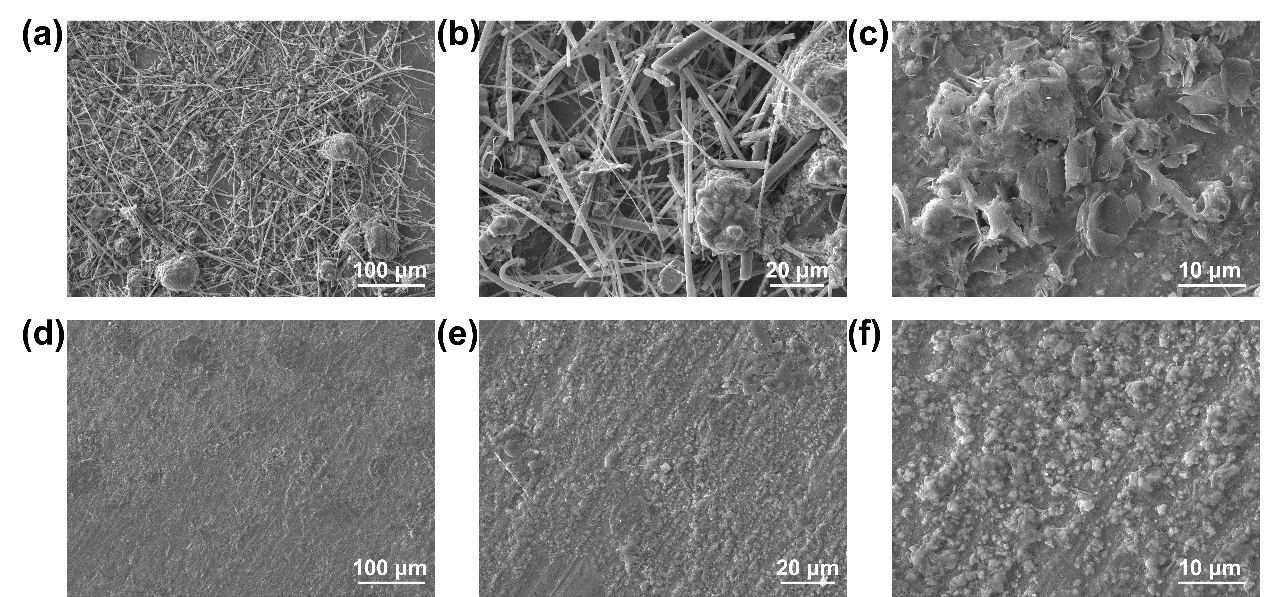


**Fig. S26** SEM images of the first deposition on the BareZn anode with the magnification of **a** 500 times, **b** 2000 times, and **c** 5000 times. SEM images of the first deposition on the CUR@Zn anode with the magnification of **d** 500 times, **e** 2000 times, and **f** 5000 times


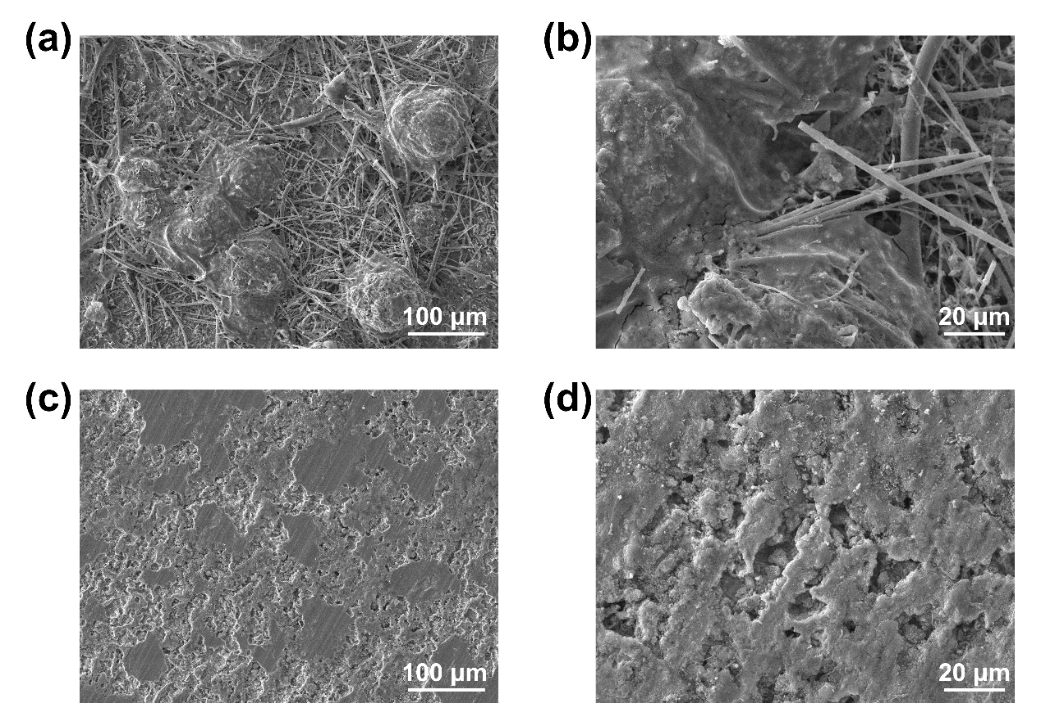


**Fig. S27** SEM images of BareZn anode after cycling for 10 cycles with the magnification of **a** 500 times, and **b** 2000 times. SEM images of CUR@Zn anode after cycling for 10 cycles with the magnification of **c** 500 times, and **d** 2000 times


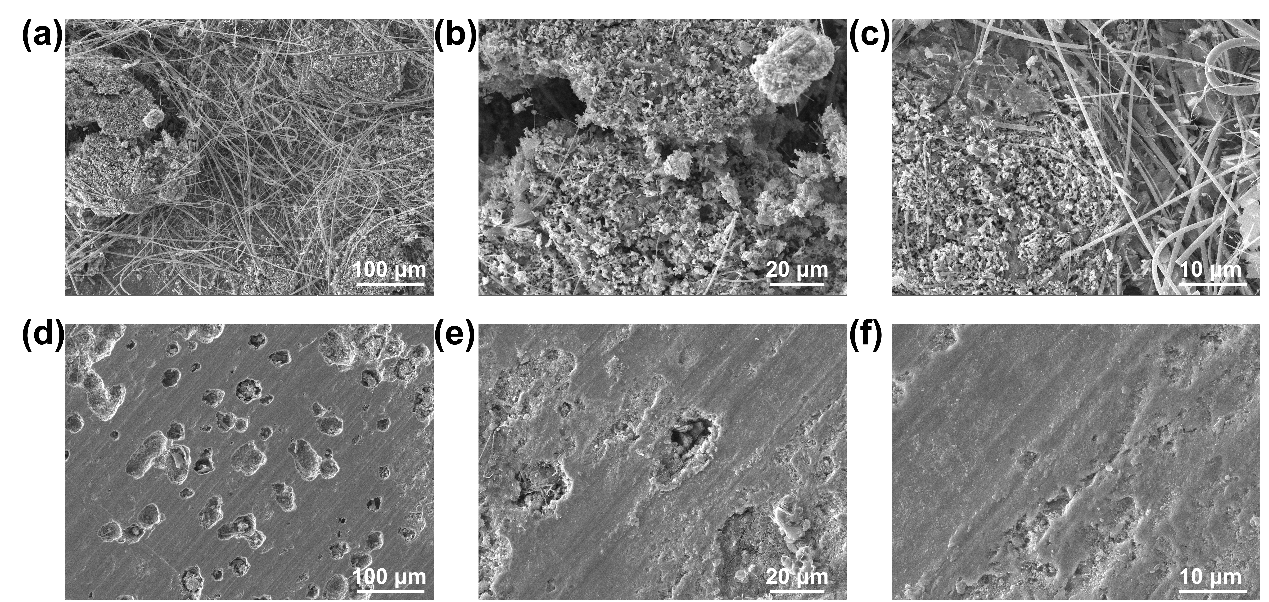


**Fig. S28** SEM images of the BareZn anode after 30 cycles with the magnification of **a** 500 times, **b** 2000 times, and **c** 5000 times. SEM images of the CUR@Zn anode after 30 cycles with the magnification of **a** 500 times, **b** 2000 times, and **c** 5000 times


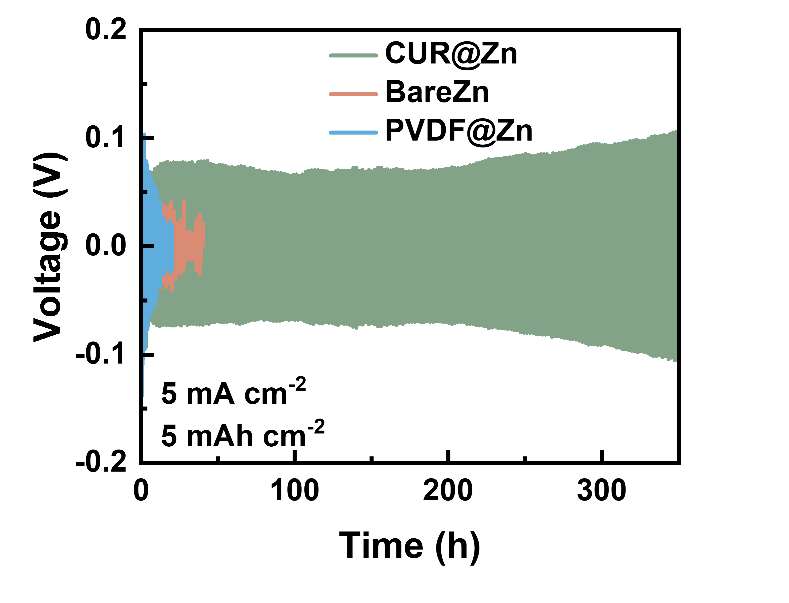


**Fig. S29** Voltage profiles of Zn||Zn symmetrical cells with different Zn foils at 5 mA cm^-2^, 5 mAh cm^-2^


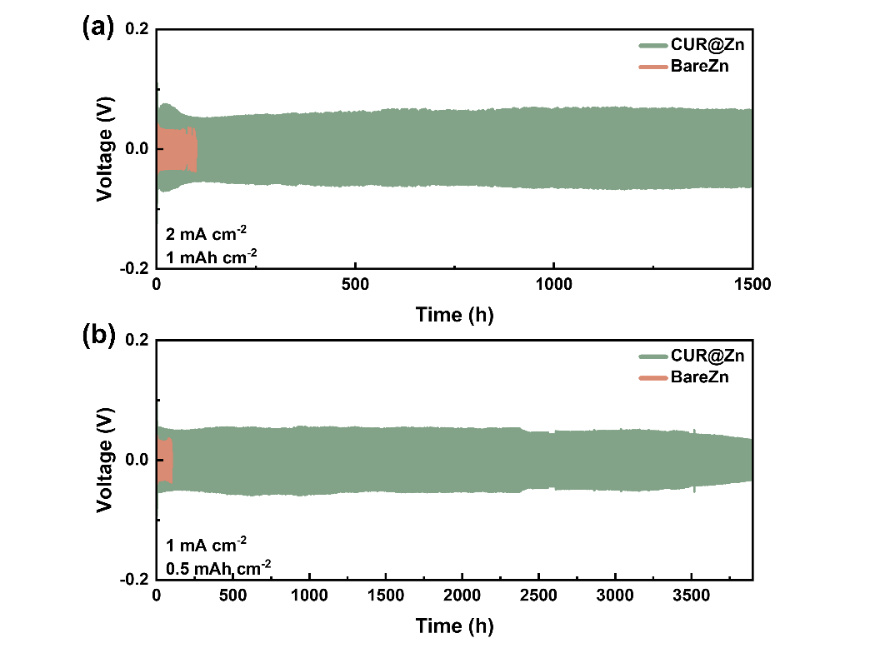


**Fig. S30** Voltage profiles of Zn||Zn symmetrical cells with different Zn foils at **a** 2 mA cm^-2^, 1 mAh cm^-2^, and **b** 1 mA cm^-2^, 0.5 mAh cm^-2^


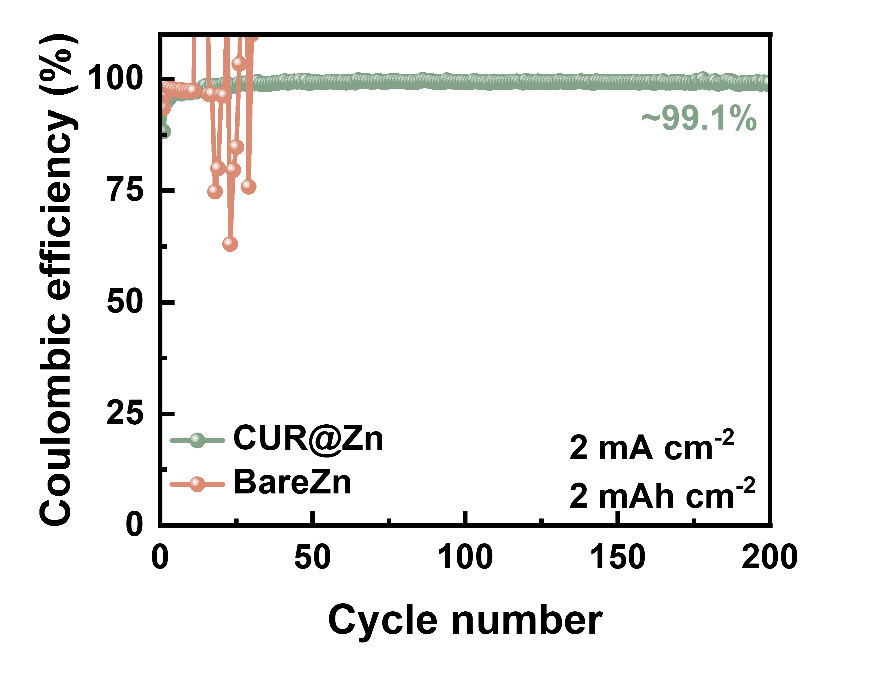


**Fig. 31** CE curves of Zn||Cu cells at 2 mA cm^-2^, 2 mAh cm^-2^


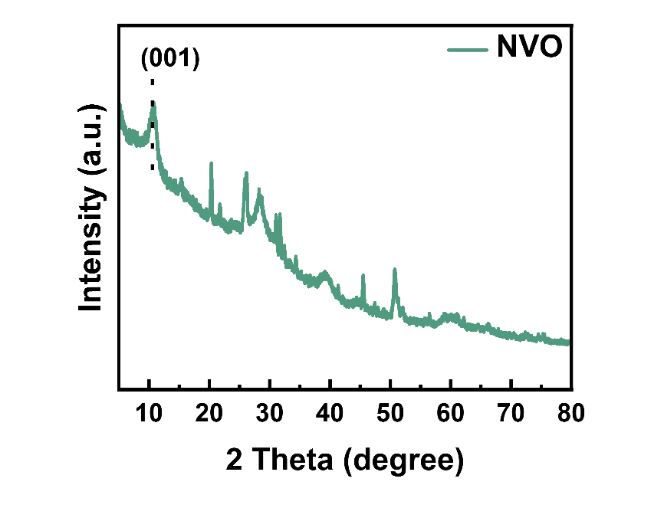


**Fig. 32** XRD pattern of the NVO powder


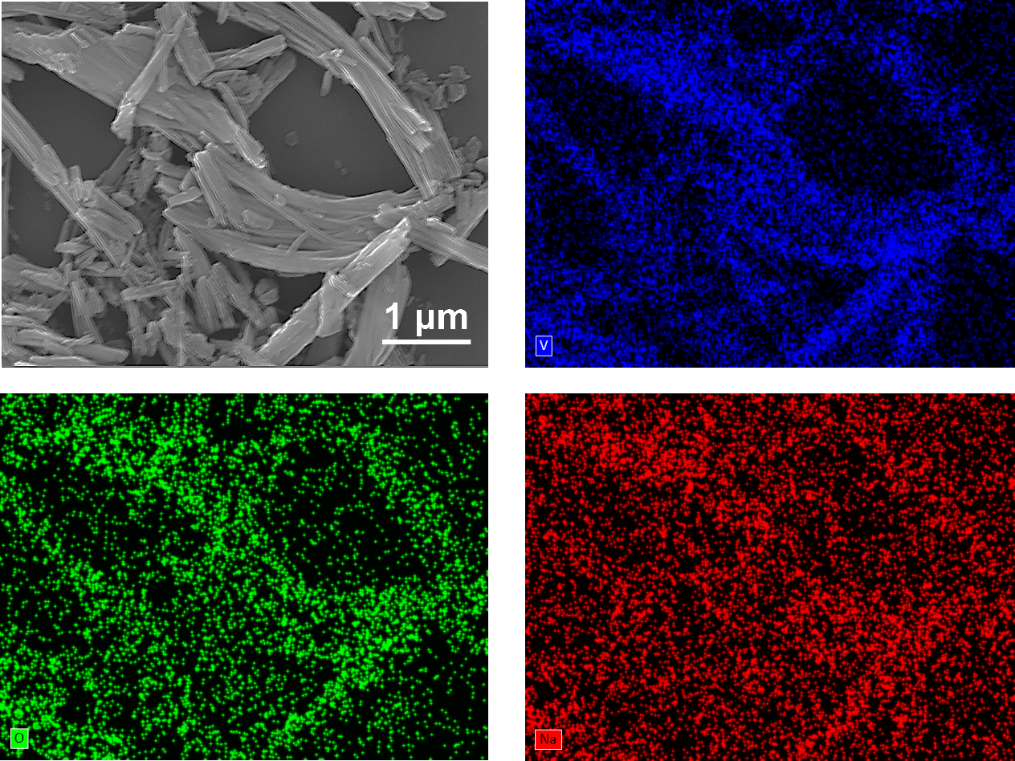


**Fig. S33** SEM image of the NVO powder and corresponding elemental mapping image


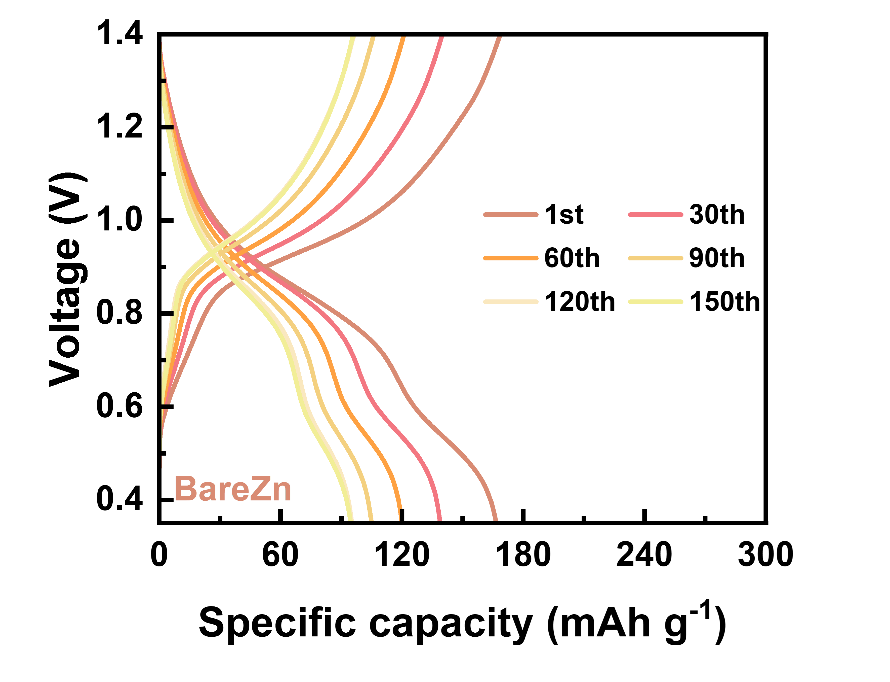


**Fig. S34** Charge-discharge curves of BareZn||NVO battery at 0.1 A g^-1^


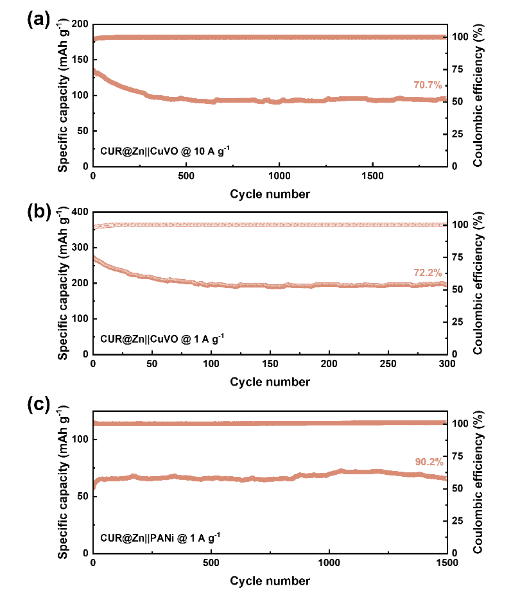


**Fig. S35** Cycling performance of CUR@Zn||CuVO batteries **a** at 10 A g^-1^, and **b** at 1 A g^-1^. **c** Cycling performance of CUR@Zn||PANi battery at 1 A g^-1^

**S3 Supplementary Table**

**Table S1** Phase-field simulation parameters

| **Parameters** | **Symbol** | **Value** | **Refs.** |
| --- | --- | --- | --- |
| Interfacial mobility | *L_σ_* | 2.7 × 10^-7^ m^3^ J^-1^ s^-1^ | [S11] |
| Kinetic coefficient | *L_η_* | 0.001 s^-1^ | [S11] |
| Electrons transferred | *n* | 2 | - |
| Transfer coefficient | *α* | 0.5 | [S12] |
| Initial electrolyte concentration | *c_0_* | 1 × 10^3^ mol m^-3^ | - |
| Site density of electrolyte | *c_s_* | 5.652 × 10^4^ mol m^-3^ | [S11] |
| Conductivity of the electrode | *σ^e^* | 10^7^ S m^-1^ | [S12] |
| Conductivity of electrolyte | *σ^s^* | 4.38 S m^-1^ | Measured |
| Diffusivity of zinc ion | *D* | 3.68 × 10^-10^ m^2^ s^-1^ | [S13] |
| Temperature | *T* | 298.15 K | Ambient |

**Table S2** Comparison of the performance of Zn plating/stripping stability in published works employing the artificial protective layer

|  | **Electrolyte** | **Current density (mA cm^-2^)** | **Areal capacity (mAh cm^-2^)** | **Life(h)** | **Accumulated cycling capacity (Ah cm^-2^)** | **Coulombic efficiency** | **Scalability ^a)^** | **Refs.** |
| --- | --- | --- | --- | --- | --- | --- | --- | --- |
| CUR@Zn | 1 M Zn(OTf)_2_ | 5 | 5 | 370 | 1.85 | 99.15% | A | This Work |
|  |  | 1 | 1 | 2000 | 2 |  |  |  |
| Zn-doped MgF_2_ | 1 M Zn(OTf)_2_ | 1 | 1 | 500 | 0.5 | - | N | [S14] |
| PANZ@Zn | 2 M Zn(OTf)_2_ | 1 | 1 | 1145 | 1.145 | 99.8% | N | [S15] |
| PVB@Zn | 1 M ZnSO_4_ | 0.5 | 0.5 | 2200 | 1.1 | 99.4% | N | [S16] |
| In@Zn | 2 M ZnSO_4_ | 0.2 | 0.2 | 1500 | 0.3 | - | N | [S17] |
| ZnF_2_@Zn | 2 M ZnSO_4_ | 1 | 1 | 800 | 0.8 | 99.5% | N | [S18] |
| C@Zn | 2 M ZnSO_4_ | 1 | 1 | 400 | 0.4 | - | A | [S19] |
| PFPE@Zn | 1 M ZnSO_4_ | 0.25 | 0.25 | 750 | 0.1875 | 99.2% | N | [S20] |
| Cu@Zn | 3 M ZnSO_4_ | 1 | 0.5 | 1500 | 1.5 | 91.8% | A | [S21] |
| ZnTAPP-NTCDA-POP@Zn | 3 M ZnSO_4_ | 0.5 | 0.5 | 1200 | 0.6 | 99.6% | A | [S22] |
| ZrO_2_@Zn | 2 M ZnSO_4_ | 0.5 | 0.125 | 3800 | 1.9 | 99.36% | N | [S23] |
| Pencil graphite@Zn | 2 M ZnSO_4_ | 0.1 | 0.1 | 200 | 0.02 | ~ 100% | N | [S24] |
| ZIF-7@Zn | 2 M ZnSO_4_ | 0.5 | 0.5 | 3000 | 1.5 | - | N | [S25] |
| NanoAu@Zn | 3 M ZnSO_4_ | 0.25 | 0.05 | 2000 | 0.5 | 97.08% | N | [S26] |
| β-PVDF@Zn | 2 M ZnSO_4_ | 0.25 | 0.05 | 2000 | 0.5 | 96.5% | N | [S27] |
| Na_3_V_2_(PO_4_)_3_@Zn | 2 M ZnSO_4_ | 0.5 | 0.25 | 500 | 0.25 | - | N | [S28] |
| F-TiO_2_@Zn | 3 M ZnSO_4_ | 2 | 2 | 280 | 0.56 | - | N | [S29] |
| CuN@Zn | 2 M ZnSO_4_ | 0.5 | 0.5 | 800 | 0.4 | - | N | [S30] |
| Nb_2_O_5_@Zn | 2 M ZnSO_4_ | 1 | 0.5 | 1000 | 1 | 98.01% | N | [S31] |
| Zn-BTC@Zn | 2 M ZnSO_4_ | 1 | 1 | 800 | 0.8 | - | N | [S32] |
| Sb_2_O_3_@Zn | 2 M ZnSO_4_ | 1 | 0.5 | 1000 | 1 | - | N | [S33] |

a). The scalability was evaluated for the applicable (A) and the non-applicable (N). If there were digital pictures of scaled-up protected zinc foils and/or the performance of the pouch cell in related publications, the scalability of the strategy would be applicable; if not, it would be non-applicable.

**Table S3** Comparison of the performance of full batteries in published works employing the artificial protective layer

| **Anode** | **Cathode** | **Electrolyte** | **Current density (A g^-1^)** | **Capacity retention** | **Ref.** |
| --- | --- | --- | --- | --- | --- |
| CUR@Zn | NVO | 1 M Zn(OTf)_2_ | 0.1 | 71.8% after 150 cycles | **This Work** |
|  |  |  | 10 | 86.5% after 3000 cycles |  |
| CuZn_5_@Zn | NMO | 2 M ZnSO_4_+0.2 M MnSO_4_ | 1 | 82.6% after 1500 cycles | [S34] |
| Nafion/Zn_3_(PO_4_)_2_@Zn | MnO_2_ | 1 M ZnSO_4_+0.1 M MnSO_4_ | 0.8 | 72.8% after 500 cycles | [S35] |
| iCOF-ED@Zn | V_2_O_5_ | 3 M Zn(OTf)_2_ | 2 | 80.8% after 1130 cycles | [S36] |
| PAN-TS@Zn | MnO_2_ | 2 M ZnSO_4_+1 M MnSO_4_ | 0.3 | 81.1% after 500 cycles | [S37] |
| MET-6@Zn | KVOH | 2 M ZnSO_4_ | 8 | 82% after 1250 cycles | [S38] |
| ZnTAPP-NTCDA-POP@Zn | NHVO | 3 M ZnSO_4_ | 1 | ~45% after 325 cycles | [S39] |
| SFM@Zn | NHVO | 2 M ZnSO_4_ | 2 | 70.64% after 1000 cycles | [S40] |
| DLC/Sn-DLC/Zn | Mn_3_O_4_-CNTs | 2 M ZnSO_4_+0.2 M MnSO_4_ | 2 | 88% after 5800 cycles | [S41] |
| CNC@AP-Zn | V_2_O_5_ | 2 M Zn(OTf)_2_ | 1 | ~37% after 1500 cycles | [S42] |
| cPANZ@Zn | MnO_2_ | 2 M ZnSO_4_+0.2 M MnSO_4_ | 1 | 90% after 300 cycles | [S43] |
| PEI-PSSNa@Zn | PTO | 2 M ZnSO_4_ | 5 | 71.2% after 1300 cycles | [S44] |
| ZPPO@Zn | V_2_O_5_ | 2 M ZnSO_4_ | 5 | 90.5% after 1500 cycles | [S45] |
| F-CDs@Zn | NHVO | 2 M ZnSO_4_ | 1 | ~58% after 500 cycles | [S46] |
| ZWO@Zn | V_6_O_13_ | 2 M ZnSO_4_ | 1 | 80% after 1000 cycles | [S47] |
| OSA-PAM@Sn-Zn | MnO_2_ | 2 M ZnSO_4_+0.1 M MnSO_4_ | 1 | 52.2% after 1000 cycles | [S48] |

**Supplementary References**

1. C. Adamo, V. Barone, Toward reliable density functional methods without adjustable parameters: The PBE0 model. J. Chem. Phys. **110**(13), 6158–6170 (1999). <https://doi.org/10.1063/1.478522>
2. F. Weigend, R. Ahlrichs, Balanced basis sets of split valence, triple *Zeta* valence and quadruple *Zeta* valence quality for H to Rn: Design and assessment of accuracy. Phys. Chem. Chem. Phys. **7**(18), 3297 (2005). <https://doi.org/10.1039/b508541a>
3. S. Grimme, S. Ehrlich, L. Goerigk, Effect of the damping function in dispersion corrected density functional theory. J. Comput. Chem. **32**(7), 1456–1465 (2011). <https://doi.org/10.1002/jcc.21759>
4. A.V. Marenich, C.J. Cramer, D.G. Truhlar, Universal solvation model based on solute electron density and on a continuum model of the solvent defined by the bulk dielectric constant and atomic surface tensions. J. Phys. Chem. B **113**(18), 6378–6396 (2009). <https://doi.org/10.1021/jp810292n>
5. G. Kresse, J. Furthmüller, Efficiency of ab-initio total energy calculations for metals and semiconductors using a plane-wave basis set. Comput. Mater. Sci. **6**(1), 15–50 (1996). <https://doi.org/10.1016/0927-0256(96)00008-0>
6. G. Kresse, J. Hafner, *Ab initio*molecular dynamics for open-shell transition metals. Phys. Rev. B **48**(17), 13115–13118 (1993). <https://doi.org/10.1103/physrevb.48.13115>
7. G. Kresse, J. Hafner, *Ab initio* molecular-dynamics simulation of the liquid-metal-amorphous-semiconductor transition in germanium. Phys. Rev. B **49**(20), 14251–14269 (1994). <https://doi.org/10.1103/physrevb.49.14251>
8. J.P. Perdew, K. Burke, M. Ernzerhof, Generalized gradient approximation made simple. Phys. Rev. Lett. **77**(18), 3865–3868 (1996). <https://doi.org/10.1103/physrevlett.77.3865>
9. P.E. Blöchl, Projector augmented-wave method. Phys. Rev. B **50**(24), 17953–17979 (1994). <https://doi.org/10.1103/physrevb.50.17953>
10. S. Grimme, J. Antony, S. Ehrlich, H. Krieg, A consistent and accurate *ab initio* parametrization of density functional dispersion correction (DFT-D) for the 94 elements H-Pu. J. Chem. Phys. **132**(15), 154104 (2010). <https://doi.org/10.1063/1.3382344>
11. Z. Hong, Z. Ahmad, V. Viswanathan, Design principles for dendrite suppression with porous polymer/aqueous solution hybrid electrolyte for Zn metal anodes. ACS Energy Lett. **5**(8), 2466–2474 (2020). <https://doi.org/10.1021/acsenergylett.0c01235>
12. L. Chen, H.W. Zhang, L.Y. Liang, Z. Liu, Y. Qi et al., Modulation of dendritic patterns during electrodeposition: a nonlinear phase-field model. J. Power Sources **300**, 376–385 (2015). <https://doi.org/10.1016/j.jpowsour.2015.09.055>
13. D.A. Cogswell, Quantitative phase-field modeling of dendritic electrodeposition. Phys. Rev. E **92**, 011301 (2015). <https://doi.org/10.1103/physreve.92.011301>
14. J.Y. Kim, G. Liu, R.E.A. Ardhi, J. Park, H. Kim et al., Stable Zn metal anodes with limited Zn-doping in MgF(2) interphase for fast and uniformly ionic flux. Nanomicro Lett. **14**(1), 46 (2022). <https://doi.org/10.1007/s40820-021-00788-z>
15. P. Chen, X. Yuan, Y. Xia, Y. Zhang, L. Fu et al., An artificial polyacrylonitrile coating layer confining zinc dendrite growth for highly reversible aqueous zinc-based batteries. Adv. Sci. **8**(11), 2100309 (2021). <https://doi.org/10.1002/advs.202100309>
16. J. Hao, X. Li, S. Zhang, F. Yang, X. Zeng et al., Designing dendrite-free zinc anodes for advanced aqueous zinc batteries. Adv. Funct. Mater. **30**(30), 2001263 (2020). <https://doi.org/10.1002/adfm.202001263>
17. D. Han, S. Wu, S. Zhang, Y. Deng, C. Cui et al., A corrosion-resistant and dendrite-free zinc metal anode in aqueous systems. Small **16**(29), 2001736 (2020). <https://doi.org/10.1002/smll.202001736>
18. Y. Yang, C. Liu, Z. Lv, H. Yang, Y. Zhang et al., Synergistic manipulation of Zn(2+) ion flux and desolvation effect enabled by anodic growth of a 3D ZnF(2) matrix for long-lifespan and dendrite-free Zn metal anodes. Adv. Mater. **33**(11), e2007388 (2021). <https://doi.org/10.1002/adma.202007388>
19. R. Yuksel, O. Buyukcakir, W.K. Seong, R.S. Ruoff, Metal-organic framework integrated anodes for aqueous zinc-ion batteries. Adv. Energy Mater. **10**(16), 1904215 (2020). <https://doi.org/10.1002/aenm.201904215>
20. S. Tao, C. Zhang, J. Zhang, Y. Jiao, M. Li et al., A hydrophobic and fluorophilic coating layer for stable and reversible aqueous zinc metal anodes. Chem. Eng. J. **446**, 136607 (2022). <https://doi.org/10.1016/j.cej.2022.136607>
21. Z. Cai, Y. Ou, J. Wang, R. Xiao, L. Fu et al., Chemically resistant Cu–Zn/Zn composite anode for long cycling aqueous batteries. Energy Storage Mater. **27**, 205–211 (2020). <https://doi.org/10.1016/j.ensm.2020.01.032>
22. X. Zhang, Y. Liu, P. Shen, L. Ren, D. Han et al., Engineering an artificial coating layer of metal porphyrin-based porous organic polymers toward high stable aqueous zinc-ion batteries. Adv. Funct. Mater. **34**(33), 2400032 (2024). <https://doi.org/10.1002/adfm.202400032>
23. P. Liang, J. Yi, X. Liu, K. Wu, Z. Wang et al., Highly reversible Zn anode enabled by controllable formation of nucleation sites for Zn-based batteries. Adv. Funct. Mater. **30**(13), 1908528 (2020). <https://doi.org/10.1002/adfm.201908528>
24. Z. Li, L. Wu, S. Dong, T. Xu, S. Li et al., Pencil drawing stable interface for reversible and durable aqueous zinc-ion batteries. Adv. Funct. Mater. **31**(4), 2006495 (2021). <https://doi.org/10.1002/adfm.202006495>
25. H. Yang, Z. Chang, Y. Qiao, H. Deng, X. Mu et al., Constructing a super-saturated electrolyte front surface for stable rechargeable aqueous zinc batteries. Angew. Chem. Int. Ed. **59**(24), 9377–9381 (2020). <https://doi.org/10.1002/anie.202001844>
26. M. Cui, Y. Xiao, L. Kang, W. Du, Y. Gao et al., Quasi-isolated Au particles as heterogeneous seeds to guide uniform Zn deposition for aqueous zinc-ion batteries. ACS Appl. Energy Mater. **2**(9), 6490–6496 (2019). <https://doi.org/10.1021/acsaem.9b01063>
27. L.T. Hieu, S. So, I.T. Kim, J. Hur, Zn anode with flexible β-PVDF coating for aqueous Zn-ion batteries with long cycle life. Chem. Eng. J. **411**, 128584 (2021). <https://doi.org/10.1016/j.cej.2021.128584>
28. N. Guo, Z. Peng, W. Huo, Y. Li, S. Liu et al., Stabilizing Zn metal anode through regulation of Zn ion transfer and interfacial behavior with a fast ion conductor protective layer. Small **19**(47), 2303963 (2023). <https://doi.org/10.1002/smll.202303963>
29. Q. Zhang, J. Luan, X. Huang, Q. Wang, D. Sun et al., Revealing the role of crystal orientation of protective layers for stable zinc anode. Nat. Commun. **11**(1), 3961 (2020). <https://doi.org/10.1038/s41467-020-17752-x>
30. Z. Yang, C. Lv, W. Li, T. Wu, Q. Zhang et al., Revealing the two-dimensional surface diffusion mechanism for zinc dendrite formation on zinc anode. Small **18**(43), e2104148 (2022). <https://doi.org/10.1002/smll.202104148>
31. S. So, Y.N. Ahn, J. Ko, I.T. Kim, J. Hur, Uniform and oriented zinc deposition induced by artificial Nb_2_O_5_ Layer for highly reversible Zn anode in aqueous zinc ion batteries. Energy Storage Mater. **52**, 40–51 (2022). <https://doi.org/10.1016/j.ensm.2022.07.036>
32. Y. Wang, Y. Liu, H. Wang, S. Dou, W. Gan et al., MOF-based ionic sieve interphase for regulated Zn^2+^ flux toward dendrite-free aqueous zinc-ion batteries. J. Mater. Chem. A **10**(8), 4366–4375 (2022). <https://doi.org/10.1039/d1ta10245a>
33. P. Xiao, Y. Wu, K. Liu, X. Feng, J. Liang et al., An ultrathin inorganic molecular crystal interfacial layer for stable Zn anode. Angew. Chem. Int. Ed. **62**(40), e202309765 (2023). <https://doi.org/10.1002/anie.202309765>
34. J. Ji, Z. Zhu, H. Du, X. Qi, J. Yao et al., Zinc-contained alloy as a robustly adhered interfacial lattice locking layer for planar and stable zinc electrodeposition. Adv. Mater. **35**(20), 2211961 (2023). <https://doi.org/10.1002/adma.202211961>
35. S. Wang, Z. Yang, B. Chen, H. Zhou, S. Wan et al., A highly reversible, dendrite-free zinc metal anodes enabled by a dual-layered interface. Energy Storage Mater. **47**, 491–499 (2022). <https://doi.org/10.1016/j.ensm.2022.02.024>
36. L. Yang, Q. Ma, Y. Yin, D. Luo, Y. Shen et al., Construction of desolvated ionic COF artificial SEI layer stabilized Zn metal anode by *in situ* electrophoretic deposition. Nano Energy **117**, 108799 (2023). <https://doi.org/10.1016/j.nanoen.2023.108799>
37. Y. Du, R. Li, T. Wang, Z. Feng, H. Dong et al., Dual effect of organic/inorganic artificial protective layer to construct high-performance zinc metal anode. Chem. Eng. J. **486**, 150139 (2024). <https://doi.org/10.1016/j.cej.2024.150139>
38. C. Li, Y. Zhang, Y. Li, Y. Gao, S. Wang et al., Artificial solid electrolyte interphases with confined ion channels and zincophilic sites for high-performance Zn metal anodes. ACS Appl. Mater. Interfaces **17**(23), 34010–34020 (2025). <https://doi.org/10.1021/acsami.5c04927>
39. X. Zhang, Y. Liu, P. Shen, L. Ren, D. Han et al., Engineering an artificial coating layer of metal porphyrin-based porous organic polymers toward high stable aqueous zinc-ion batteries. Adv. Funct. Mater. **34**(33), 2400032 (2024). <https://doi.org/10.1002/adfm.202400032>
40. W. Zhou, Z. Chen, S. Zhao, S. Chen, Superhydrophobic and highly flexible artificial solid electrolyte interphase inspired by *Lotus* effect toward highly stable Zn anode. Adv. Funct. Mater. **34**(49), 2409520 (2024). <https://doi.org/10.1002/adfm.202409520>
41. X. Guo, Q. Peng, K. Shin, Y. Zheng, S. Tunmee et al., Construction of a composite Sn-DLC artificial protective layer with hierarchical interfacial coupling based on gradient coating technology toward robust anodes for Zn metal batteries. Adv. Energy Mater. **14**(38), 2402015 (2024). <https://doi.org/10.1002/aenm.202402015>
42. J. Cao, Z. Wang, Z. Yang, K. Tang, S. Wang et al., High-performance zinc anodes enabled by atmospheric plasma enhanced cellulose protective layer for zinc ion batteries. J. Power Sources **627**, 235699 (2025). <https://doi.org/10.1016/j.jpowsour.2024.235699>
43. J. Yang, S. Wang, L. Du, S. Bi, J. Zhu et al., Thermal-cyclized polyacrylonitrile artificial protective layers toward stable zinc anodes for aqueous zinc-based batteries. Adv. Funct. Mater. **34**(21), 2314426 (2024). <https://doi.org/10.1002/adfm.202314426>
44. W. Li, C. Wang, W. Hu, Y. Wang, C. Li et al., A weak acid-responsive porous polyelectrolyte membrane enables the high efficiency of a zinc anode interface. Chem. Sci. **16**(30), 13873–13882 (2025). <https://doi.org/10.1039/d5sc02326b>
45. Q. Liu, L. Jiao, J. Wang, H. Bai, C. Yi et al., Kinetics-mediating artificial interphase for ultrafast Zn metal anodes. Adv. Funct. Mater. **35**(17), 2422868 (2025). <https://doi.org/10.1002/adfm.202422868>
46. Z. Ge, L. Xu, Y. Xu, J. Wu, Z. Geng et al., Multifunctional fluorinated carbon dots artificial interface layer coupled with *in situ* generated Zn^2+^ conductor interlayer enable ultra-stable Zn anode. Nano Energy **119**, 109053 (2024). <https://doi.org/10.1016/j.nanoen.2023.109053>
47. C. Yi, L. Jiao, J. Wang, Y. Ma, H. Bai et al., Multi-scale functionally designed ZnWO_4_ artificial interphase for ultra-stable aqueous Zn metal anodes under high current rates. Adv. Funct. Mater. **34**(45), 2404579 (2024). <https://doi.org/10.1002/adfm.202404579>
48. W. Li, L. Li, X. Fu, Y. Hu, Y. Deng, Innovative design of a double-layer gradient coating for dendrite-free and ultrastable zinc anodes. Small **21**(11), e2411915 (2025). <https://doi.org/10.1002/smll.202411915>
